# A Meta-Analysis of the CSAI-2 and CSAI-2R with Objective Sport Performance from 1985–2026

**DOI:** 10.3390/sports14080334

**Published:** 2026-08-04

**Authors:** Marc Lochbaum, Fanny Arendt

**Affiliations:** 1 Department of Kinesiology and Sport Management, Texas Tech University, Lubbock, TX 79409, USA; 2 Research Institute, Education Academy, Vytautas Magnus University, 44248 Kaunas, Lithuania; 3 The Graduate School, Texas Tech University, Lubbock, TX 79409, USA

**Keywords:** CSAI-2R, pre-competitive anxiety, self-confidence, quantitative analysis, athletic performance, measurement timing, competitive athletics

## Abstract

This review provides a substantial, updated meta-analysis examining the relationships between pre-competition Competitive State Anxiety Inventory-2 (CSAI-2) and Competitive State Anxiety Inventory-2 Revised (CSAI-2R) subscales and objective sport performance metrics from 1985 to 2026. Following Preferred Reporting Items for Systematic reviews and Meta-Analyses (PRISMA) guidelines, we found studies via a systematic search of the Web of Science (WOS) and EBSCOhost databases, as well as relevant meta-analyses. Random-effects models calculated pooled mean differences (Hedges’ *g*) and correlation coefficients (r) for the primary analyses. Forty-eight studies totaling 3659 athletes met the inclusion criteria. The included literature using primarily the CSAI-2 spanned four decades of global research, featuring mainly individual sport elite and university-level samples of male and female athletes. Primary findings revealed significant pooled effects in the hypothesized directions across all subscales in both the mean difference and correlation studies, ranging from small to medium in magnitude. Prediction intervals were wide for all primary analyses, indicating high variability in future study findings. Of note, subgroup datasets for win/lose comparisons in tennis and martial arts, golf and gymnastics event scores, and individual athlete’s basketball and volleyball statistics provided more stable results providing direction for researchers and practitioners. In conclusion, while the CSAI-2(R) remains a popular pre-competitive monitoring tool, wide prediction intervals except in a few subgroups necessitate caution. Practitioners should prioritize measurement of the CSAI-2(R) the day of performance with longitudinal, idiographic assessments to account for individual zones of optimal functioning and anxiety interpretation.

## 1. Introduction

More than two decades have passed since meta-analyses by Craft et al. [1] and Woodman and Hardy [2] examined the relationship between pre-competitive anxiety and self-confidence with measures of sport performance. Craft and colleagues, with only CSAI-2 [3] data, reported non-significant correlations for both anxiety dimensions, while Woodman and Hardy identified a small negative relationship between cognitive anxiety and performance. Regarding self-confidence and performance, both meta-analyses reported a small significant relationship. Despite minimal quantitative support and CSAI-2 criticisms prior to both meta-analyses being published [4,5], research utilizing the CSAI-2, as well as with the CSAI-2R [6], continued. Therefore, this review aims to provide a substantial update of the peer-reviewed CSAI and sport performance research literature.

### 1.1. The CSAI-2

Anxiety and sport performance is a popular historic sport psychology topic, as evidenced by published meta-analyses [7]. Kleine [8] published the first broadly defined anxiety and sport performance meta-analysis including 50 studies with publication dates between 1970 and 1988. Kleine provided evidence across state and trait anxiety measures that anxiety is related to sport performance. Jokela and Hanin’s [9] meta-analysis of 19 studies from 1978 to 1997 provided support for the ability of the IZOF to discriminate between successful and less successful athletes based on state anxiety scores. A major shift in sport psychology research occurred with the introduction of the Multidimensional Theory of Anxiety by Martens et al. [3], which replaced unidimensional inverted-U models with three separate subcomponents: cognitive anxiety, somatic anxiety, and self-confidence. Central to this shift was the development of the 27-item CSAI-2 (see the Appendix A.1), designed to assess how athletes feel “right now” prior to competition. The CSAI-2 rapidly became the measure of choice in sport anxiety and self-confidence research. Another major shift that came out of Multidimensional Theory was that cognitive anxiety and self-confidence are independent, orthogonal factors rather than endpoints of a single continuum. In addition, Martens and colleagues hypothesized a curvilinear relationship between somatic anxiety and performance, though Woodman and Hardy [2] questioned the rationale supporting a curvilinear relationship.

Two meta-analyses [1,2], both published in 2003, examined both the pre-competitive anxiety and self-confidence literature. With only CSAI-2 samples, Craft and colleagues [1] synthesized 29 studies that included both athletes and physical education students. They found weak effect-size relationships among the anxiety subscales and performance and a small effect for self-confidence (self-confidence: *r* = 0.25; cognitive anxiety: *r* = 0.01; somatic anxiety: *r* = −0.03). The authors noted that the hypothesized curvilinear relationship for somatic anxiety might explain its non-significant singular finding, though few published manuscripts have evaluated this hypothesis. Woodman and Hardy [2] reported small and significant relationships with sport performance for both self-confidence (*r* = 0.24) and cognitive anxiety (*r* = −0.10), with mostly CSAI-2 samples. Of importance, they reported that the relationship between pre-competitive self-confidence and performance was significantly smaller with the CSAI-2 than other confidence measures. The small relationship between pre-competitive self-confidence and sport performance has been verified in two more recent meta-analyses [10,11].

Both Craft and her colleagues [1] and Woodman and Hardy [2] explored potential moderators, but these results were inconsistent in measurement and results. Craft et al. found that CSAI-2 states were more strongly related to performance in athletes participating in individual sports rather than team sports. Stronger relationships also appeared for open-skill tasks and when the inventory was administered between 31 and 59 min before competition. However, the authors cautioned against their exploratory modeling approach due to testing for moderation. Woodman and Hardy identified competitive standard and participant sex as significant moderators. Stronger relationships for cognitive anxiety and self-confidence emerged in higher-standard compared to lower-standard competitions and in samples consisting of 100% males compared to females. Lochbaum et al. [11] reaffirmed the moderating effect of sex, reporting a significant difference between 100% male and 100% female samples for self-confidence.

Despite limited quantitative support for the CSAI-2 in relation to sport performance and purported psychometric shortcomings [4,5], the original instrument remains widely used in sport psychology literature, even with the CSAI-2R being published in 2003 [6] (see the Appendix A.2). Cox and colleagues performed a two-stage Confirmatory Factor Analysis (CFA) process to address the factor-structure limitations of the original scale. An initial analysis of 503 intramural athletes indicated an inadequate model fit, leading to the scale being trimmed to 17 items by removing problematic cross-loading variables. When this revised model was subsequently assessed on a separate group of 331 competitive athletes, it achieved statistical validity. Consequently, the resulting CSAI-2R should be the preferred measure for both practical and research applications. In addition to the CSAI-2R not being the preferred measure, there is little evidence in the CSAI literature that the importance of a directional component to the measurement of the CSAI scales [4] took hold, as post hoc analysis of the included studies in this systematic review found only one study since 2010 by León-Prados et al. [12] measuring athletes’ facilitative and debilitative perceptions. Likewise, few studies have evaluated the curvilinear relationship between somatic anxiety and performance proposed by Martens and colleagues. As with the directional component, only León-Prados and colleagues have reported on the evaluation of the curvilinear relationship between somatic anxiety and performance since 2010.

### 1.2. Study Purposes

Given the continued interest in cognitive anxiety, somatic anxiety, and self-confidence as measured by the CSAI-2 and CSAI-2R, this review provides an updated meta-analysis of pre-competition CSAI-2(R) with objective sport performance. A critical update in relation to Craft et al. [1] and Woodman and Hardy [2] involves the inclusion of only studies with objective sport performance measures (e.g., event score, race times, game statistics, or group differences such as winning or losing), as opposed to including self-reported performance or other such measures in the literature (e.g., performance satisfaction and coach-rated performance). We intend to examine moderators based on Terry’s propositions [13] and the four relevant meta-analyses [1,2,10,11], such as performance outcomes, scale version (CSAI-2 or CSAI-2R), participant demographics, and the timing of pre-competitive CSAI measurements, if the included studies provide sufficient data.

## 2. Materials and Methods

We conducted this systematic review and meta-analysis in accordance with the PRISMA 2020 guidelines [14]. The study protocol was pre-registered and is publicly available through the Open Science Framework (https://doi.org/10.17605/OSF.IO/ZTRNX). Reference management and screening of database search results were executed using Rayyan Cambridge, MA, USA (https://new.rayyan.ai/ (26 December 2025)). All meta-analytic statistical procedures were performed using Comprehensive Meta-Analysis (CMA) Version 4 Englewood, NJ, USA [15,16,17].

### 2.1. Literature Search and Article Management

We conducted the primary literature search on 26 December 2025 and subsequently updated for self-confidence on 16 February 2026. The following databases were searched in both instances: WOS (Social Science Citation Index, Emerging Sources Citation Index, and Science Citation Index Expanded) and EBSCOhost (APA PsycArticles, ERIC, Medline, Psychology and Behavioral Sciences Collection, PsycINFO, and SPORTDiscus).

The primary search targeted title, keyword, and abstract fields for studies relating to the following measures and terms: Competitive State Anxiety Inventory-2, CSAI-2, Revised Competitive State Anxiety Inventory-2, CSAI-2R, Sport Anxiety Scale, SAS, Sport Anxiety Scale-2, SAS-2, somatic anxiety, cognitive anxiety, and concentration disruption. The string of measures and terms was connected with AND sport and the following terms with NOT: music, physical education, physical activity, academics, randomized control trial, clinical trials, meta-analysis, systematic review, scoping review, sleep disorder, clinical depression, anxiety disorder, concussion, cancer, injury, retrospective. The NOT terms were utilized based on an initial search of WOS and EBSCOhost to best delimit the search to relevant articles. In WOS, results were filtered to human subjects and original research articles to align with the inclusion criteria. Although the initial search included the Sport Anxiety Scale (SAS/SAS-2), the lack of relevant performance data led to an exclusive focus on the CSAI-2(R) literature.

The supplemental search in both WOS and EBSCOhost, conducted on 16 February 2026, covered January 2021 to February 2026, with the intent to capture more recent self-confidence data potentially missed by the confidence meta-analyses of Jekauc et al. [10] and Lochbaum et al. [11], utilizing the following string: Confidence AND (CSAI-2 ORCSAI-2R) AND sport performance.

### 2.2. Screening and Selection Process

**Figure 1:**
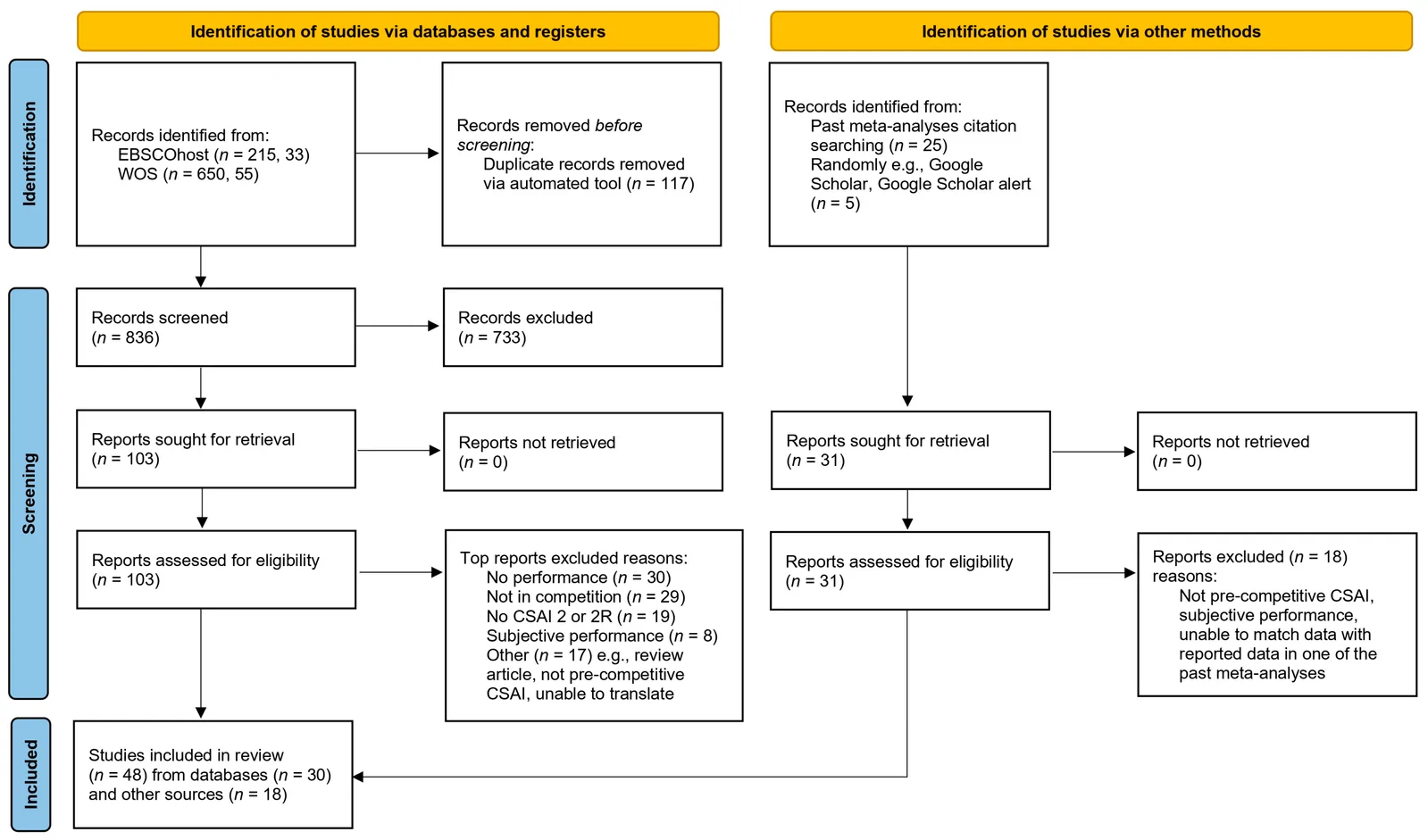
PRISMA flow chart depicting the search strategy for the included studies [4,12,18,19,20,21,22,23,24,25,26,27,28,29,30,31,32,33,34,35,36,37,38,39,40,41,42,43,44,45,46,47,48,49,50,51,52,53,54,55,56,57,58,59,60,61,62,63].

Following the removal of duplicates in Rayyan on 26 December 2026, then again on 16 February 2026, both authors, blind to each other’s decisions in Rayyan, screened the remaining titles and abstracts. Discrepancies between the two authors were resolved through consensus during weekly meetings. The identified studies were cross-referenced with the relevant meta-analyses [1,2,8,10,11]. Correspondence with authors of studies published after 2020 was attempted to obtain necessary effect-size data; however, no authors replied with data. Figure 1 details the selection process.

### 2.3. Inclusion and Exclusion Criteria

Studies were included in the meta-analysis if they met the following criteria: published in a peer-reviewed scholarly journal; used the CSAI-2 or CSAI-2R; reported that the CSAI-2/2R was administered pre-competition; conducted in a competitive sport setting, involved participants identifiable as athletes according to the Lochbaum et al. [64] classification system, used objective measures of sport performance (e.g., game outcomes, performance statistics, or race times), and could be translated into English via Google Translate.

Studies were excluded based on the following criteria: non-English manuscripts for which both authors lacked confidence in the Google Translate translation, published in gray literature (theses, dissertations, book chapters, conference abstracts/proceedings, and review papers) or utilized data already included from another study, employed measures “inspired by” the CSAI, measured the CSAI-2(R) post event, included participants falling outside the Lochbaum et al. [64] athlete classification (e.g., physical education students), and used subjective performance measures such as athlete performance self-satisfaction and coach ratings.

The following items were retrieved from each included study: sample size, mean age or age range, female percentage of the total sample, author-provided sample description, the name or description of the competition/sporting event, the specific CSAI measure used and its language for data collection, data collection procedures and the timing of the CSAI measurement relative to competition, a description of the performance measure, and the necessary statistical data for effect-size calculations. The two authors conducted data extraction collaboratively.

### 2.4. Risk-of-Bias and Publication-Bias Assessment

To assess publication bias, four distinct statistical measures were utilized: Orwin’s fail-safe *n* [65], the classic fail-safe *n* [66], funnel-plot visualization [67], and Duval and Tweedie’s [68] trim-and-fill procedure. Definitions and explanations of each risk-of-bias statistical measure, as well as all others used in this meta-analysis, are presented in the Supplemental Files (Table S1). To evaluate the risk of bias across the individual studies, we used a modified version of Hoy and colleagues’ [69] risk-of-bias tool (Table S2). Though Hoy et al. designed their measure to assess risk of bias in prevalence studies across 10 items and one summary item with a maximum score of 10, their measure has since been adapted in pre-competition sport psychology meta-analyses [11,70] their items assessing both internal and external validity appear to have been useful in developing a similar or inspired by measure. In these past meta-analyses, three levels of assessment per rated item, as opposed to Hoy and colleagues’ 10-point system, were developed by the study authors. In the present meta-analysis, we worked through iterations to develop the three levels of statements for each of Hoy and colleagues’ 10 items. Point values (high, medium, and low) were assigned to each item, and a study quality score (range: 9 to 30 points) was computed, where a higher score indicates a lower risk of bias. For instance, for Hoy item 8, “Was the same mode of data collection used for all participants?”, we assigned 3 points for “Yes, same mode for all participants”, 2 points for “Unsure or appeared self-regulated by participants”, and 1 point for “No, a mix of modes such as coach collected some, research staff collected some of the CSAI data”. For Hoy item 10, “Were the numerator(s) and denominator(s) for the parameter of interest appropriate?”, we assigned 3 points for “Multiple CSAI and multiple performance measures”, 2 points for “One CSAI and one performance measure”, and 1 point for “One CSAI with multiple performances”. All assigned points were finalized through consensus between the two study authors.

### 2.5. Effect-Size Calculations and Analysis

Mean values (within or between groups) and correlation coefficients were expected to be the primary datasets. Studies reporting multiple outcomes for the same metric were pooled to one effect-size value. Studies reporting mean difference and correlation data were entered separately into the appropriate mean difference or correlational datasets. Both sets of data were entered to reflect self-confidence as benefitting performance and cognitive and somatic anxiety as hindering performance. Thus, for the correlation data, attention was given to the direction of the relationship of the outcome for the sport in question. To convert median data to mean data, the method proposed by Wan and colleagues [71] was used for Conde-Ripoll et al. [29] and Conde-Ripoll et al. [30]. Correlation magnitudes (*r*) were interpreted using standard conventions: 0.10 to 0.29 as small, 0.30 to 0.49 as medium, and greater than 0.50 as large. Mean differences were evaluated by calculating Hedges’ *g*, which was interpreted using standard benchmarks: 0.20 to 0.49 as small, 0.50 to 0.79 as medium, and greater than 0.80 as large.

Given the anticipated differences in athletic populations, performance settings, and performance outcomes, and given that we intend to use our results to generalize to a wider universe of athletes in performance settings, we used the random-effects model in the meta-analytic analyses. For the primary effect sizes, the following are reported: number of samples, summary statistics, 95% confidence and prediction intervals, Q-value, Tau^2^, *I*^2^, and publication-bias statistics. Statistical significance for the primary analyses was set at the traditional *p* < 0.05. The mixed-effects model was used for moderator analyses, as well as the random-effects model in meta-regression. Though there is no consensus with respect to the number of samples required per moderator category, 10 samples per moderator category are recommended for reliable estimates with diverse study samples, such as those in the present meta-analysis (Borenstein, personal communication 8 June 2026). At the outset, the possibility of meeting 10 samples per moderator category was unknown though unlikely. Given the number of moderator analyses across all three CSAI scales, greatly increasing chance findings, the *p*-value for statistical significance was set at *p* < 0.01.

Lastly, in addition to the statistics previously mentioned, to further evaluate the stability of the primary findings, a remove-one sensitivity analysis was performed to gauge the influence of individual studies on the overall effect size. A cumulative analysis by publication year was conducted to assess the consistency of the observed relationships over time.

## 3. Results

### 3.1. Study Characteristics

Forty-eight studies (see Table 1 and Table S3) totaling 3659 athletes met all inclusion criteria [4,12,20,21,22,23,24,25,26,27,28,29,30,31,32,33,34,35,36,37,38,39,40,41,42,43,44,45,46,47,48,49,50,51,52,53,54,55,56,57,58,59,60,61,62,63]. Publication dates spanned from 1985 to 2026, with the following number of articles found in each decade: 1980s, *n* = 3; 1990s, *n* = 11; 2000s, *n* = 7; 2010s, *n* = 10; and 2020s, *n* = 17. Two studies—those of Burton [26] and León-Prados et al. [14]—reported on different athlete samples at different sporting events, while Terry et al. [57] reported on the same participants competing in both singles and doubles tennis. Given the two distinct samples at different sport events—those of Burton and León-Prados et al.—we listed them as Study 1 and Study 2 in the meta-analytic analyses, whereas data from Terry et al. (1996) [57] were combined as one effect size per CSAI scale. Both the CSAI-2 (*n* = 38) and CSAI-2R (*n* = 10) were used in the included studies in various languages other than English, including Chinese, Croatian, English, Estonian, French, Flemish, Greek, Hungarian, Italian, Portuguese, and Spanish. Nearly all the studies reported extractable data for cognitive anxiety, somatic anxiety, and self-confidence.

Data collections across the 48 included studies from local competitions to world championships with primarily elite (*n* = 16) or advanced (*n* = 15) athletes. Most samples consisted of adults and adolescents, with only two youth samples. Sporting events were primarily individually based (over 70% of studies), with golf (*n* = 5), martial arts (*n* = 5), gymnastics (*n* = 4), tennis (*n* = 4), and swimming (*n* = 4) most represented. Volleyball (both beach and traditional variants) was the most sampled team sport (*n* = 4). Participant samples were male-only (*n* = 24), with the remainder being mixed (*n* = 14) or female-only (*n* = 11). Regarding measurement timing, 25 studies collected data within 60 min of performance, 14 collected data on the event day more than 60 min prior, and 8 collected data the day before competition.

Twenty-four studies provided mean-difference data between performance groups (e.g., winners vs. losers, medalists vs. non-medalists). Winners compared with. losers was the most frequent comparison (*n* = 14). Kenshloo et al. [39] reported a won/lost point biserial correlation with the CSAI-2 subscales; thus, their study was included among the mean-difference studies. Two studies [27,45] compared outcomes within the same teams. Thirty studies provided correlational data for at least one CSAI scale. Performance metrics included race times, event placement, scores, and individual in-game statistics.

### 3.2. Risk of Bias Within Studies

The risk-of-bias scores for all samples entered within CMA were assessed using our tool adapted from Hoy et al. [69], yielding a mean quality score of 22.21 (*SD* = 1.89), a median score of 22.00, and a range of 18 to 25. Meta-regression analyses found that the risk-of-bias scores did not significantly account for variance in either the mean-difference or correlational meta-analyzed data. Full risk ratings are located in the Supplemental Files (Table S4).

### 3.3. Synthesis of Mean Study Data

**Figure 2:**
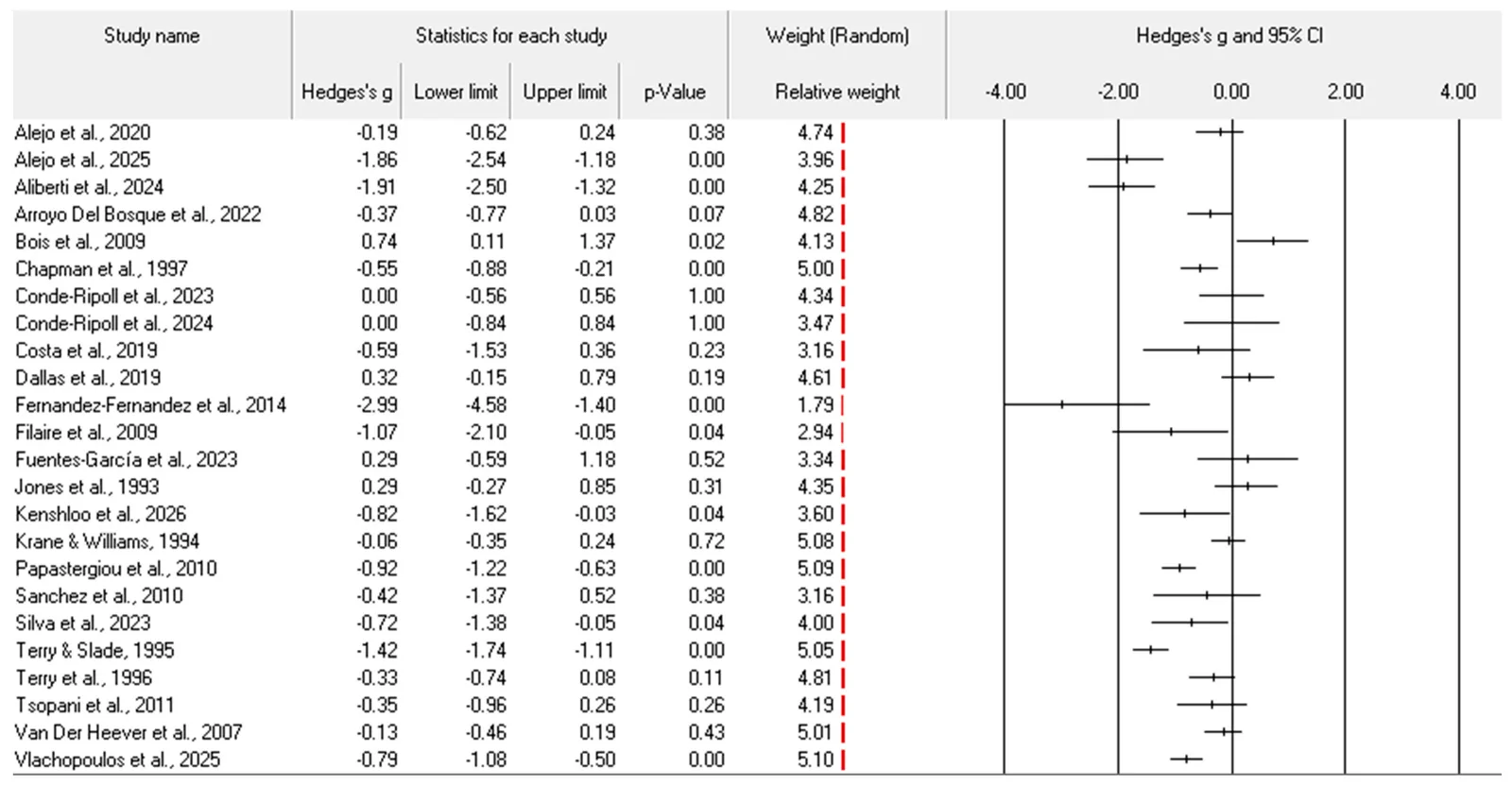
Cognitive anxiety studies in order [4,18,19,20,21,25,27,28,29,30,31,32,33,34,35,39,40,49,53,54,58,59,60,61]. Effect-size statistics expressed as Hedges’ *g* with corresponding forest-plot samples organized by outcome. Data to the left of 0.00 indicates cognitive anxiety was lower in the higher-performing groups, while data to the right of 0.00 indicates cognitive anxiety was higher in the higher-performing groups. The red bars indicate a visual representation of the relative weight of each study.

**Figure 3:**
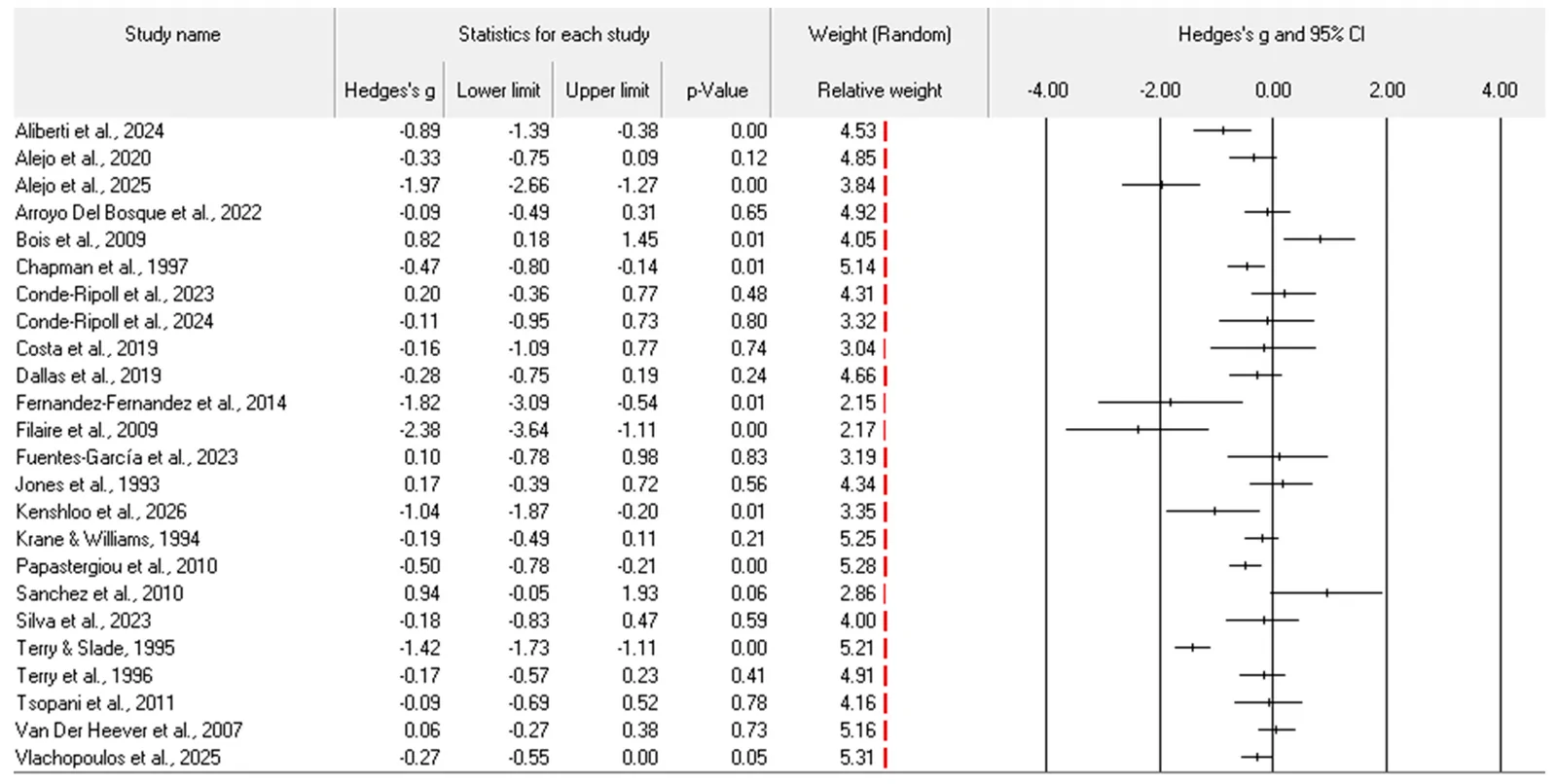
Somatic anxiety studies in order [4,18,19,20,21,25,27,28,29,30,31,32,33,34,35,39,40,49,53,54,58,59,60,61]. Effect-size statistics expressed as Hedges’ *g* with corresponding forest-plot samples organized by outcome. Data to the left of 0.00 indicates somatic anxiety was lower in the higher-performing groups, while data to the right of 0.00 indicates somatic anxiety was higher in the higher-performing groups. The red bars indicate a visual representation of the relative weight of each study.

**Figure 4:**
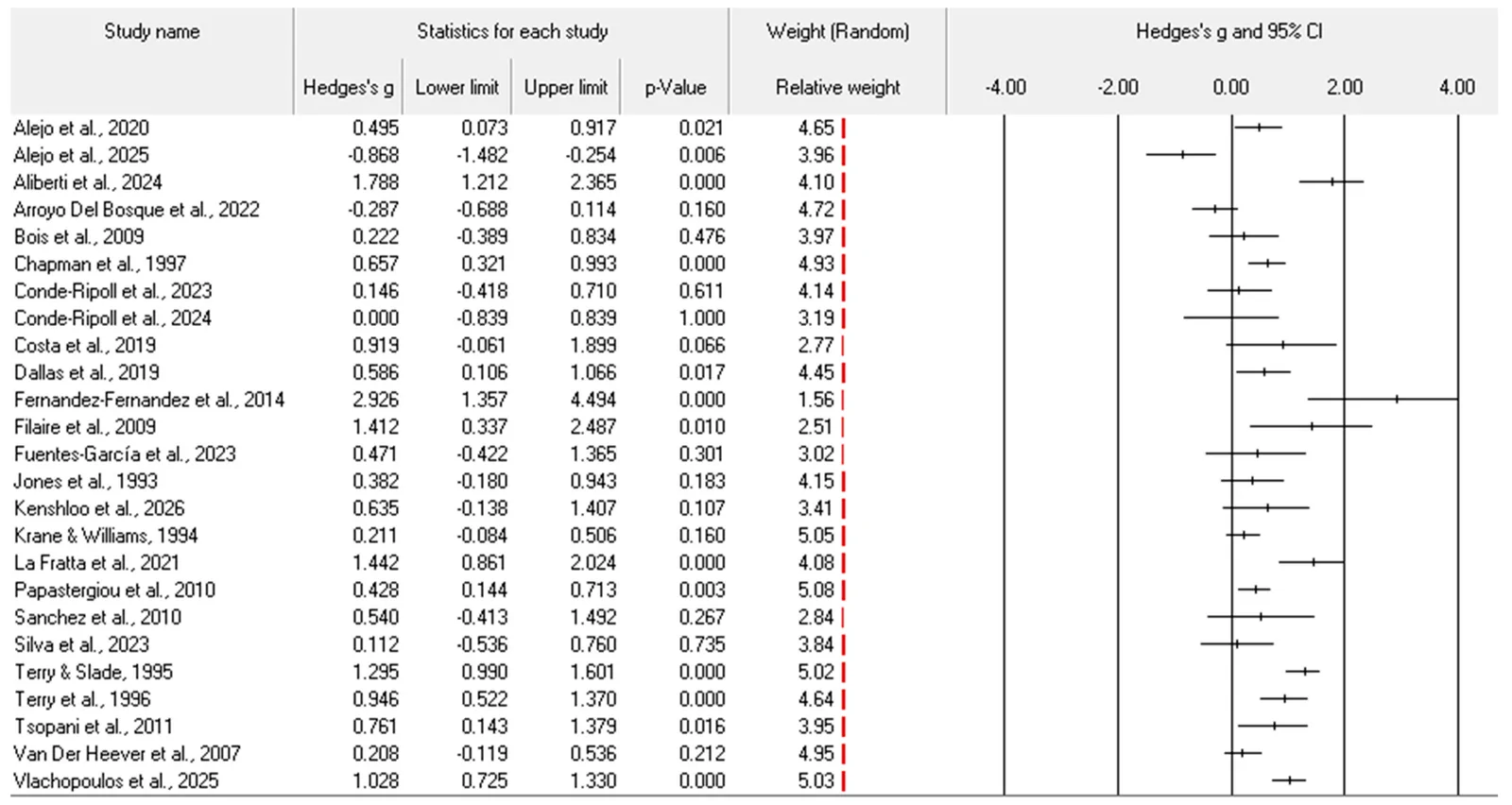
Self-confidence studies in order [4,18,19,20,21,25,27,28,29,30,31,32,33,34,35,39,40,41,49,53,54,58,59,60,61]. Effect-size statistics expressed as Hedges’ *g* with corresponding forest-plot samples organized by outcome. Data to the left of 0.00 indicates self-confidence was lower in the higher-performing groups, while data to the right of 0.00 indicates self-confidence was higher in the higher-performing groups. The red bars indicate a visual representation of the relative weight of each study.

The individual effect-size data for cognitive anxiety, somatic anxiety, and self-confidence are presented in Figure 2, Figure 3 and Figure 4. Even with the variety of participants, sports, and defined outcome groupings, most effect sizes aligned with the hypothesized directions of cognitive and somatic anxiety hindering performance and self-confidence benefiting performance. Several studies contributed exceptionally large effect-size values, all in the hypothesized direction, for at least two of the primary analyses [19,20,33,34,58]. Two studies reported within-team mean data [27,45]. The individual study figures for those studies are located to the Supplemental Materials (Figure S1).

**Figure 5:**
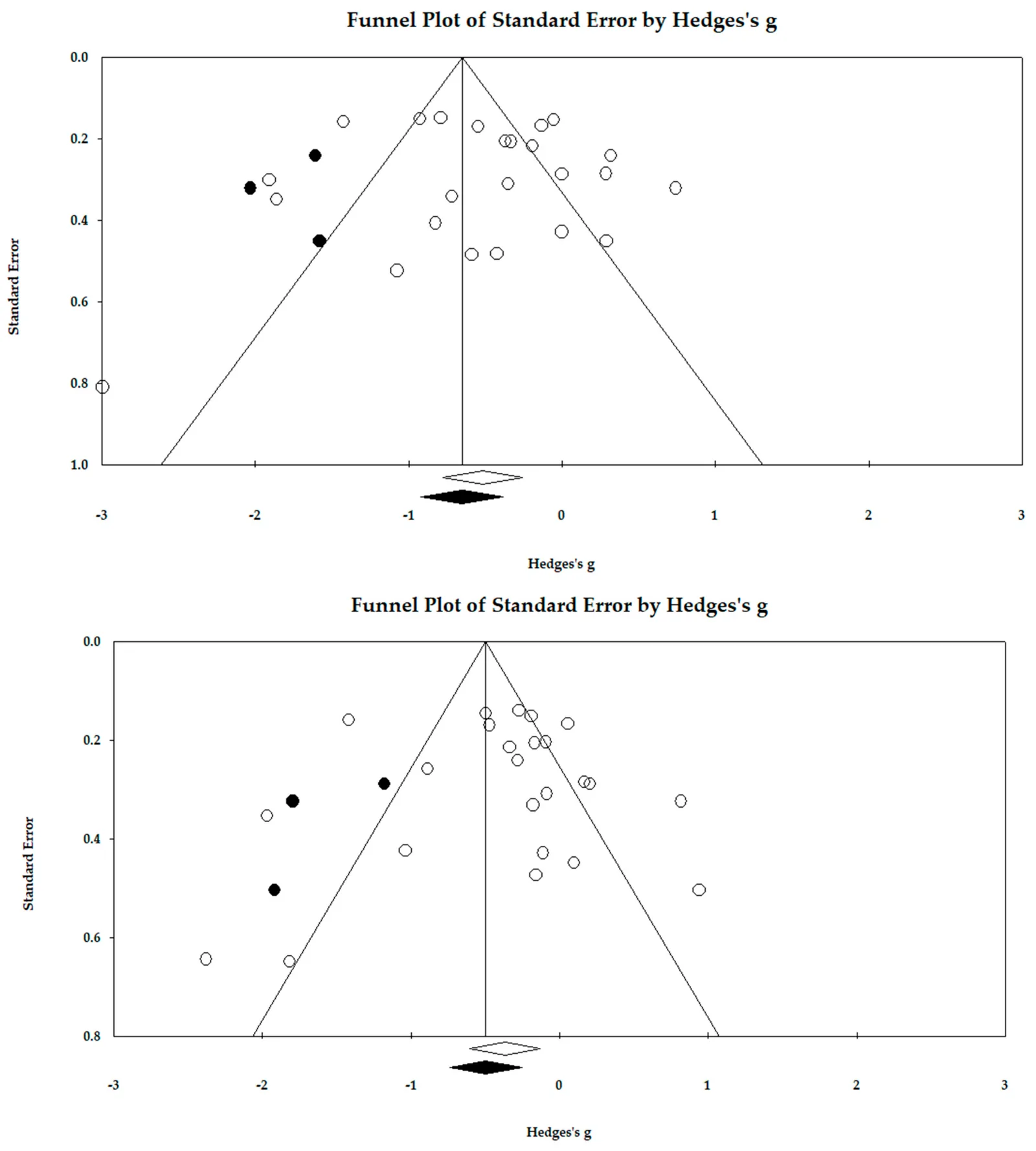

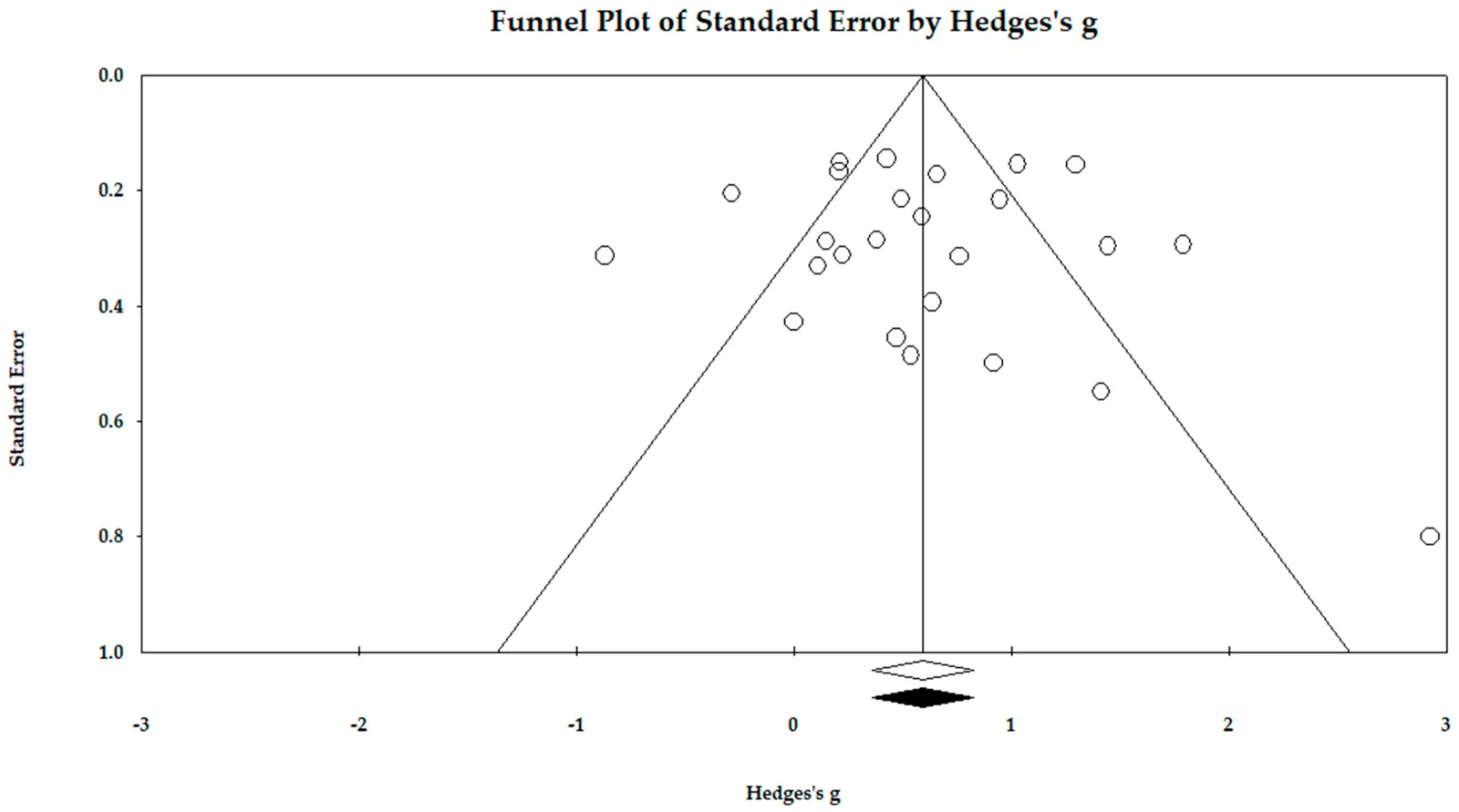
Random-effects trim-and-fill plots for cognitive anxiety studies [4,18,19,20,21,25,27,28,29,30,31,32,33,34,35,39,40,49,53,54,58,59,60,61] (**top** figure), somatic anxiety studies [4,18,19,20,21,25,27,28,29,30,31,32,33,34,35,39,40,49,53,54,58,59,60,61] (**middle** figure), and self-confidence studies in order [4,18,19,20,21,25,27,28,29,30,31,32,33,34,35,39,40,41,49,53,54,58,59,60,61] (**bottom** figure). The open circles are the data points. The black circles are trimmed and filled data points. The clear rhombus is the mean effect size. The black rhombus is the trimmed and filled mean effect size.

Cognitive anxiety and self-confidence *g* values demonstrated medium effect sizes, while the somatic-anxiety effect size was small (see Table 2). All three CSAI-subscale 95% confidence intervals excluded zero, indicating statistical significance. While the 95% confidence intervals were significant, the 95% prediction intervals for each scale encompassed zero, suggesting variability in results in comparable future samples of higher- and lower-performing athletes, supporting the need for moderator or subgroup analyses. Figures illustrating these intervals are included in the Supplemental Files (Figure S1). Based on the Q-statistic, heterogeneity was present for each primary analysis. Based on the included studies, publication bias for the CSAI scales appeared minimal, though the trim-and-fill analyses for cognitive anxiety (*g* = −0.65 [−0.92, −0.39]) and somatic anxiety (−0.50 [−0.74, −0.26]) suggested noticeable shifts to account for influential studies with large positive effect sizes. The funnel plots are presented in Figure 5. Regarding the two within-team studies [27,45], all mean effect-size values were negligible, and none of the Hedges’ *g* values in these specific samples reached statistical significance (cognitive anxiety: *g* = −0.03 [−0.34, 0.28]; somatic anxiety: *g* = −0.15 [−0.45, 0.16]; self-confidence: *g* = 0.14 [−0.17, 0.44]; see Supplemental Figure S2 for individual data and the corresponding forest plot).

### 3.4. Mean-Difference Moderator Results

Though none of the mixed-effects analyses were significant at *p* < 0.01, significant individual effect-size data from subgroups within the performance category and timing of the CSAI measurement prior to performance resulted at *p* < 0.01. It is critical to point out that, except for two individual effects, the prediction intervals still spanned from hindering to benefiting performance for each CSAI scale.

Table 3 contains the CSAI scales by performance outcomes grouped into one of the four following categories: individual results (e.g., placed or medaled), researcher-defined outcomes (e.g., good/bad performance), win/loss in an individual contest, and win/loss in a team contest. In the primary analysis (data located in Table 2), we included only one effect size per CSAI scale per study and did so for the moderator analyses. Two studies provided two different performance-category datasets [59,61], and Terry et al. [57] provided two events with the same sample. Those datasets were excluded from the moderator sport performance analyses.

The win/loss individual effect sizes were significantly different than 0 (*p* < 0.01) for all three CSAI scales. Though still encompassing 0, the prediction intervals ranged from minimal to hindering winning for somatic anxiety and from minimal to benefiting winning for self-confidence, whereas the cognitive anxiety prediction intervals ranged from hindering winning to benefiting winning. Lastly, for the sport performance mean-difference results, for self-confidence, the effect size of the researcher-defined performance-outcome category was significant (*p* < 0.01) for self-confidence, though the prediction intervals were wide.

Along with the performance categories, the CSAI timing results provided significant (*p* < 0.01) individual effect-size values. Specifically, for CSAI assessment 60 min or less prior to performance, the effect sizes were statistically different than 0 (*p* < 0.01), with comparable results with “day of” measurement timing for cognitive anxiety and somatic anxiety. For self-confidence, the effect sizes for 60 min or less and the “day before” assessment were more dependable than the day-of data. Importantly, the prediction intervals for the timing results were wide for all measurement subgroups and, thus, provided doubt as to the best time of pre-competition assessment.

### 3.5. Mean-Difference Sensitivity Results

To further evaluate the stability of the primary findings, sensitivity analyses, including “remove-one” analysis and cumulative analysis by year, were conducted (see Supplemental Figures S3 and S4). Even with large effect-size values from studies, the “remove-one” analyses demonstrated that no single study disproportionately influenced the overall effect-size ranges. For the cumulative analysis by year, except for the study by Bois and colleagues [25], the pattern of results in the last two decades has consistently provided evidence supporting the continued use of the CSAI-2 or 2R in relation to sport performance.

### 3.6. Synthesis of Correlation Data

**Figure 6:**
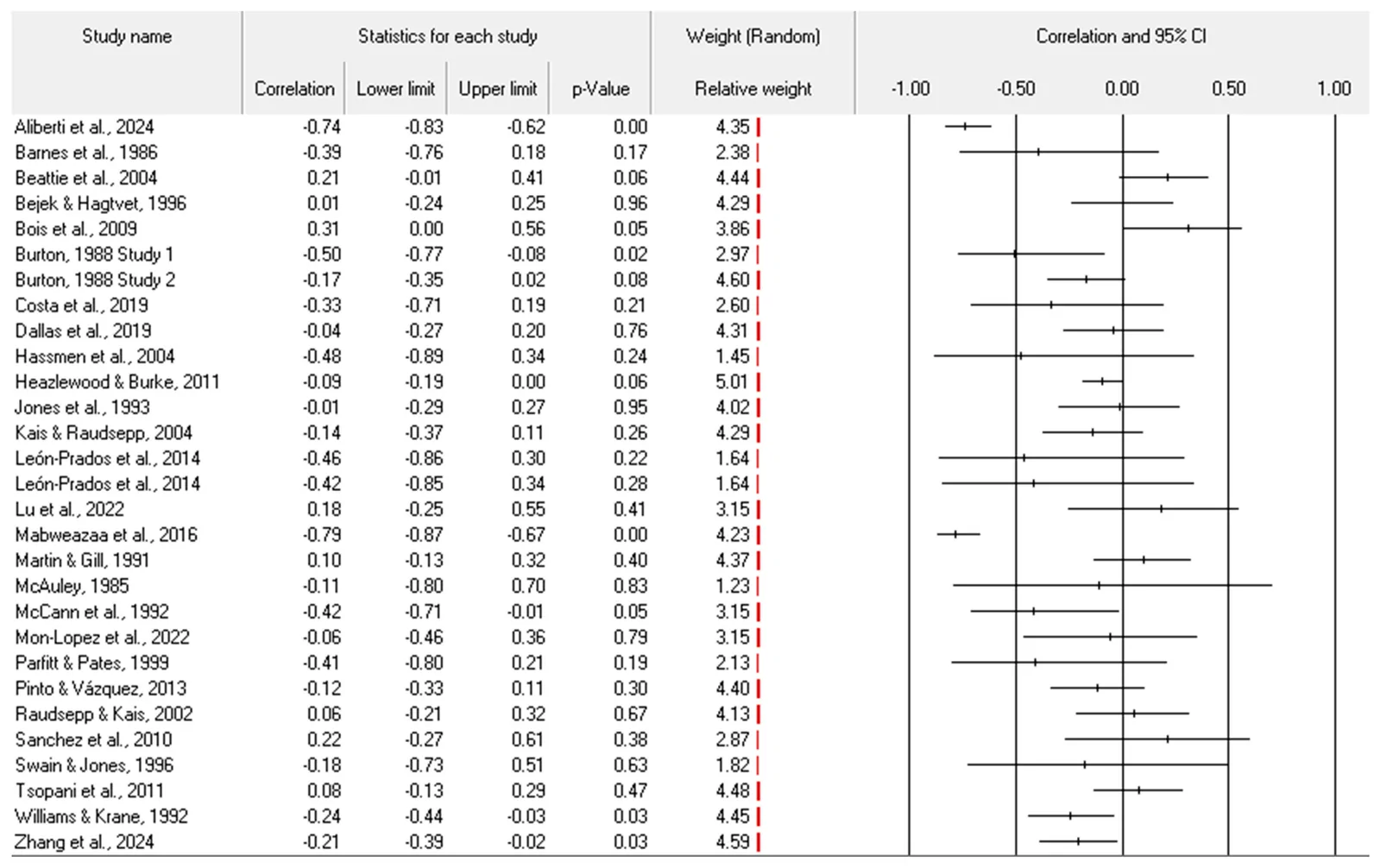
Cognitive anxiety studies in order [4,12,20,22,23,24,25,26,31,32,36,37,38,42,43,44,46,47,48,50,51,52,53,56,59,62,63]. Effect-size statistics expressed as *r* with corresponding forest-plot samples organized by outcome. Data to the left of 0.00 indicates cognitive anxiety hindered performance, while data to the right of 0.00 indicates cognitive anxiety benefited performance. The red bars indicate a visual representation of the relative weight of each study.

**Figure 7:**
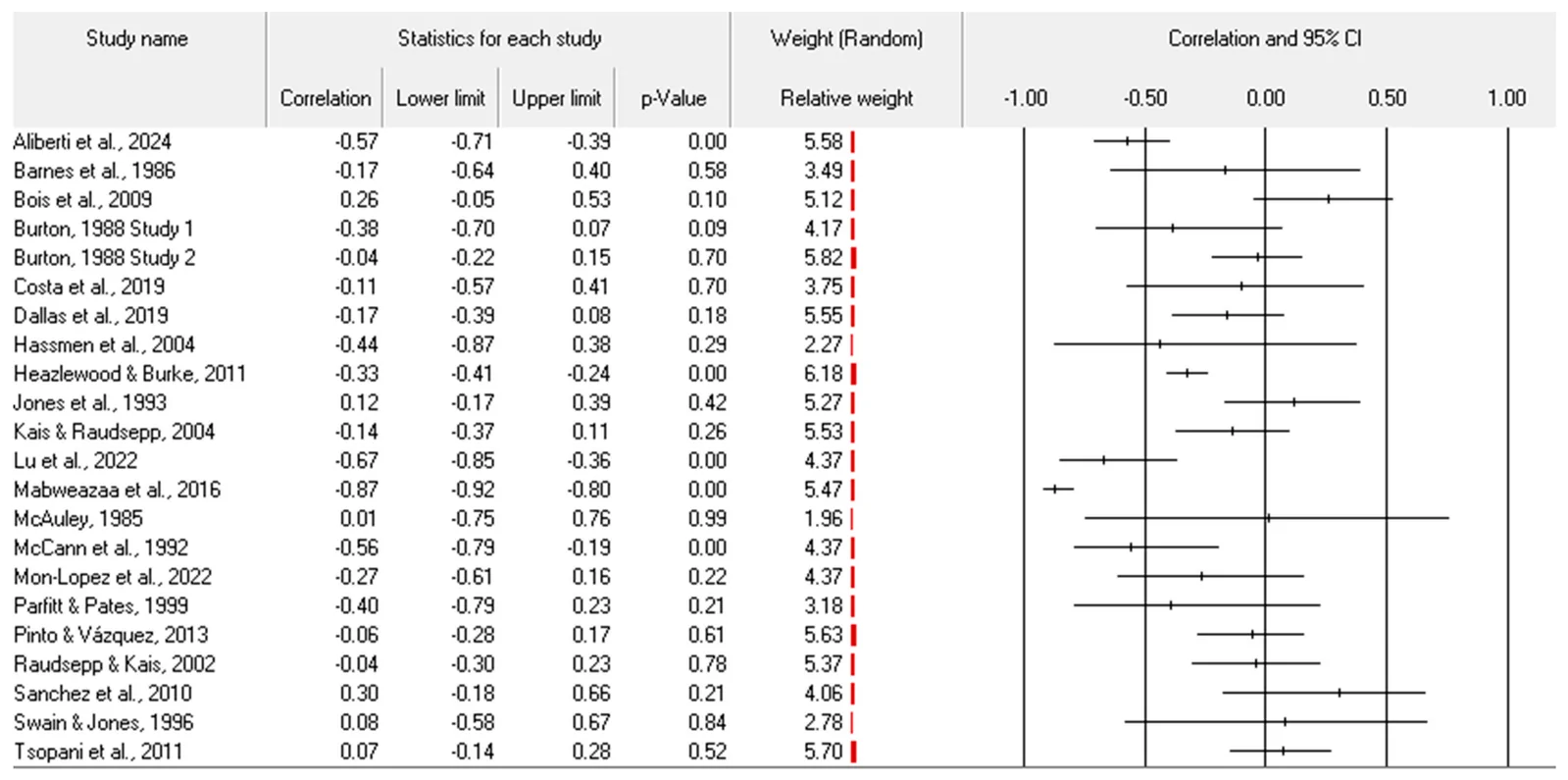
Somatic anxiety studies in order [4,20,22,25,26,31,32,36,37,38,42,43,46,47,48,50,51,52,53,56,59]. Effect-size statistics expressed as *r* with corresponding forest-plot samples organized by outcome. Data to the left of 0.00 indicates somatic anxiety hindered performance, while data to the right of 0.00 indicates somatic anxiety benefited performance. The red bars indicate a visual representation of the relative weight of each study.

**Figure 8:**
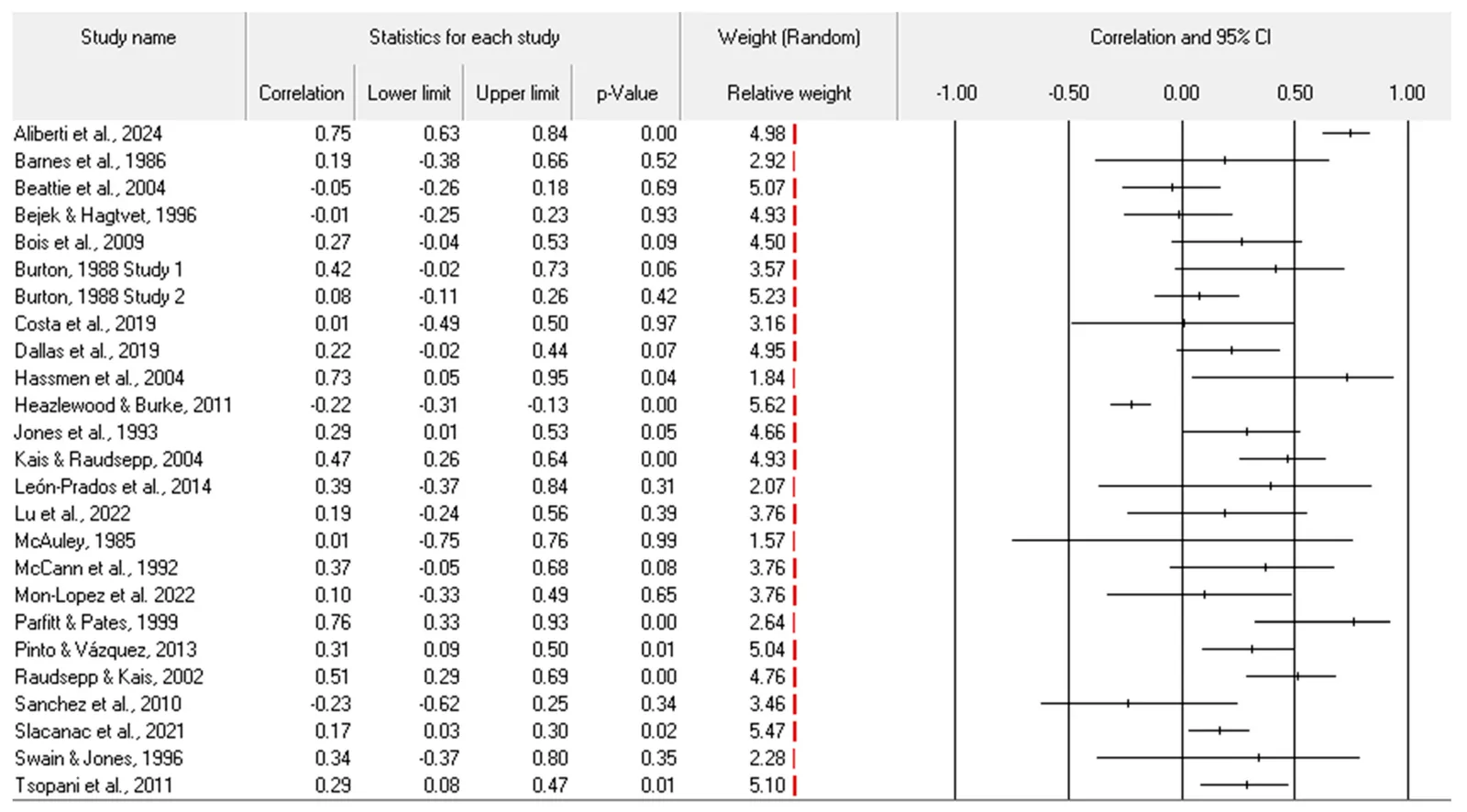
Self-confidence studies in order [4,12,20,22,23,24,25,26,31,32,36,37,38,42,46,47,48,50,51,52,53,55,56,59]. Effect-size statistics expressed as *r* with corresponding forest-plot samples organized by outcome. Data to the left of 0.00 indicate self-confidence hindered performance, while data to the right of 0.00 indicate self-confidence benefited performance. The red bars indicate a visual representation of the relative weight of each study.

Figure 6, Figure 7 and Figure 8 depict the individual study data for cognitive anxiety, somatic anxiety, and self-confidence. As with the mean-difference samples, most individual correlational effect-size values were in the hypothesized directions. Aliberti et al. [20], in their study including dancesport athletes, contributed large (*r* > 0.50) effect-size values for all three CSAI-scale analyses, along with Study 1 by Burton [26] and the study by Mabweazaa et al. [43] for cognitive anxiety; the study by McCann et al. [47] for somatic anxiety; and the studies by Hassmen et al. [36], Parfit and Pates [50], and Raudsepp and Kais [52] for self-confidence.

**Figure 9:**
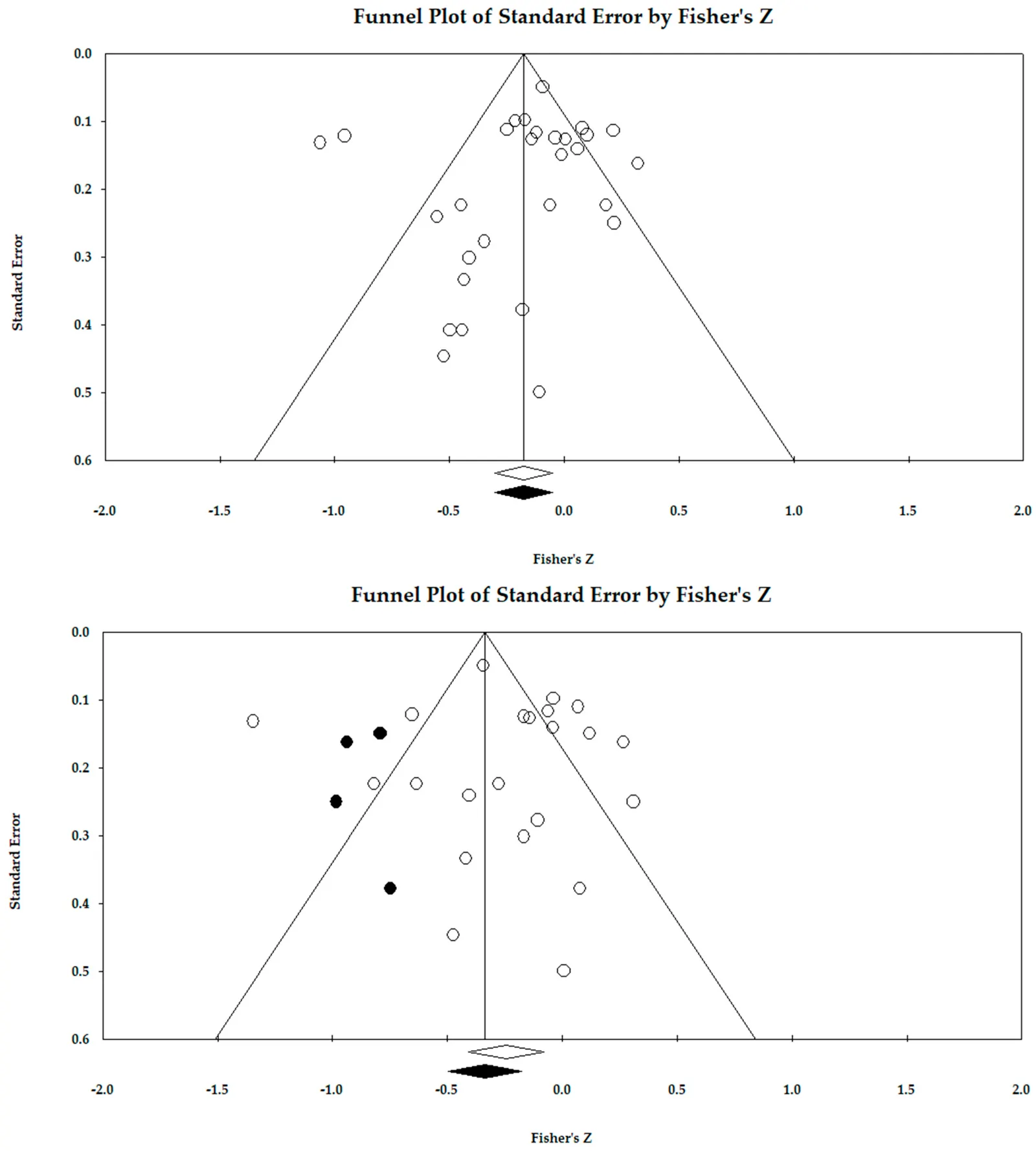

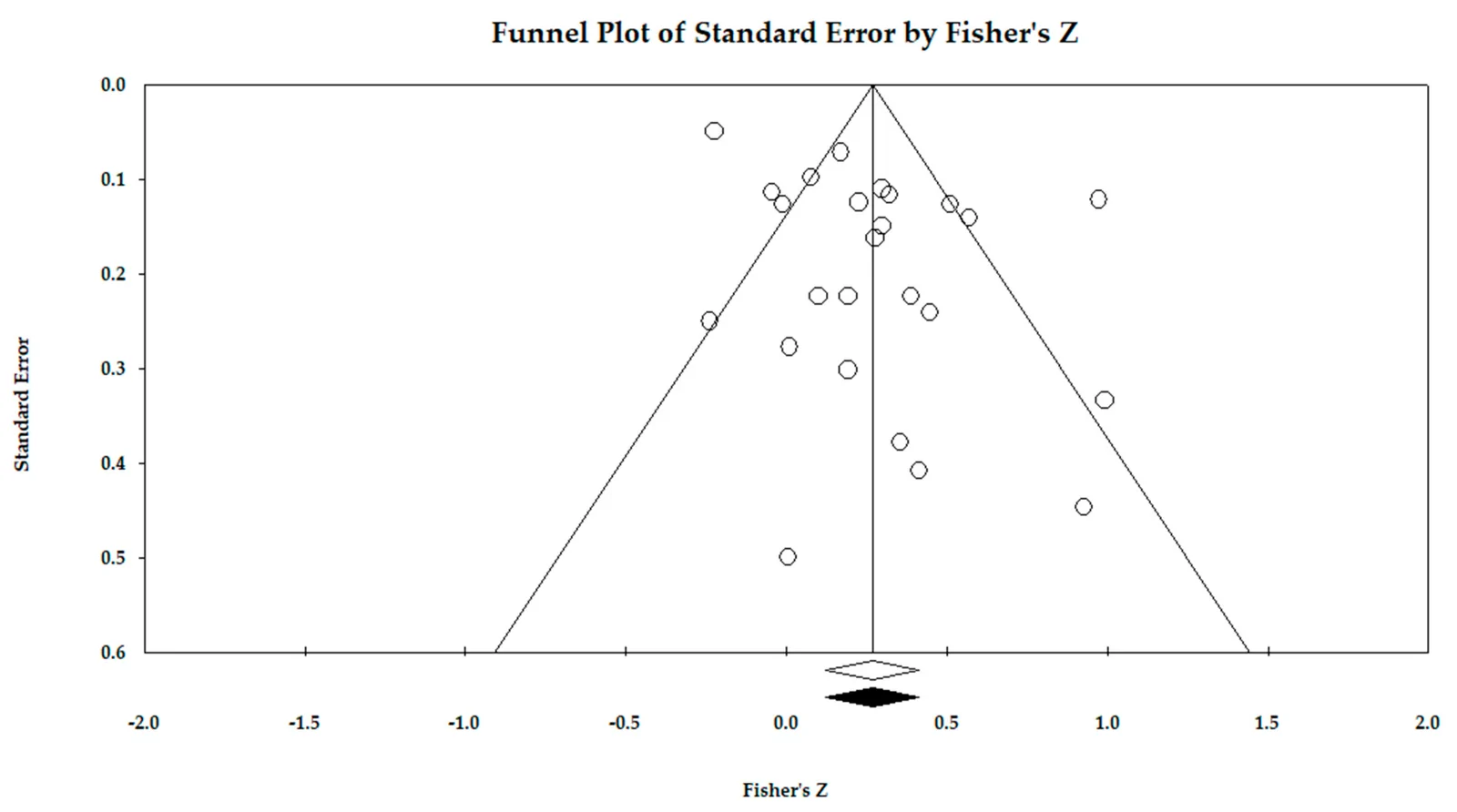
Random-effects trim-and-fill plots for cognitive anxiety studies in order [12,20,22,23,24,25,26,31,32,36,37,38,42,43,44,46,47,48,50,51,52,53,56,59,62,63] (**top** figure), somatic anxiety studies in order [20,22,25,26,31,32,36,37,38,42,43,46,47,48,50,51,52,53,56,59] (**middle** figure), and self-confidence studies [4,12,20,22,23,24,25,26,31,32,38,42,46,47,48,50,51,52,53,55,56,59] (**bottom** figure). The open circles are the data points. The black circles are trimmed and filled data points. The clear rhombus is the mean effect size. The black rhombus is the trimmed and filled mean effect size.

As presented in Table 4, the relationship between all CSAI subscales and performance was small in meaningfulness and significantly different than zero. The 95% confidence intervals indicated that the observed mean from the included studies could fall between small and medium in meaningfulness for somatic anxiety and self-confidence and range from less than small to small for cognitive anxiety. Given the methodological variety across the samples, it is not surprising that the true prediction intervals for each scale were wider than the 95% confidence intervals from the observed studies. Figures illustrating these intervals are presented in the Supplemental Files (Figure S5). As with the primary mean-difference results of the included studies, significant heterogeneity was present across all scales; however, publication bias was minimal, with only somatic anxiety showing a shift from −0.24 to −0.32 [95% CIs: −0.46, −0.17] based on the trim-and-fill analysis. The funnel plots are presented in Figure 9.

### 3.7. Correlation Moderator Results

As with the mean-difference moderator results, none of the correlation-based mixed-effects moderator analyses reached statistical significance (*p* < 0.01). Unlike the mean-difference results, subgroupings were significant (*p* < 0.01), with no heterogeneity (see Table 5). First, for objective sport performance, the following two categories were formed: individual sports objective outcomes and individual statistics from athletes performing on a team. The mean correlations were small in meaningfulness, except for the individual team self-confidence correlation that was medium in meaningfulness. Nearly all 95% confidence intervals for the pooled correlations excluded 0, though the prediction intervals did include 0.

Next, the individual sports category was separated into common measures, placement (all scores, along with golf and gymnastics, analyzed separately) race time, and race time adjusted to the individual athlete. The largest meta-analyzed correlations were for placement, with prediction intervals ranging from no relationship to a large relationship (i.e., cognitive and somatic anxiety hindering and self-confidence benefiting performance). Race-time data were inconsistent across the CSAI scales. Score data separated by the two most represented sports, golf and gymnastics, were informative in that the prediction intervals ranged from no impact to beneficial for self-confidence and from no impact to hindering for cognitive anxiety. Next, the individual basketball and volleyball statistics resulted in patterns like those for golf and gymnastics. Lastly, CSAI timing subgroups reported in Table 5. Specifically, author-stated data collected within one hour of the competition were moderate in magnitude and significantly different than 0, though the prediction intervals included 0 within a wide range.

### 3.8. Correlation Data Sensitivity Results

As with the mean-difference studies, sensitivity analyses, including “remove-one” analysis and cumulative analysis by year, were conducted for the correlation studies (Figures S6 and S7). Though variations were evident, the “remove-one” analysis demonstrated that no single study disproportionately influenced the overall effect sizes for cognitive anxiety, somatic anxiety, or self-confidence. For the cumulative analysis by year, the effect sizes showed variation over time. However, as with the mean-difference sample, the pattern of results in the last 10 years provides stronger evidence supporting the use of the CSAI in performance studies.

## 4. Discussion

The primary objective of this systematic review and meta-analysis was to provide an update to the pre-competition CSAI and sport performance literature. Unlike past anxiety and confidence meta-analyses [1,2,8,9,10,11], we only included studies with objective sport performance outcomes. By synthesizing data from 48 studies spanning 1985 to 2026, this review provides an important update regarding the pre-competitive CSAI literature with both group and correlational sport performance data. While Craft et al. [1] reported non-significant correlations for both cognitive and somatic anxiety, our primary results, except for the two within-team samples [27,45] demonstrate in our included studies that cognitive and somatic anxiety, along with self-confidence, are significantly related to objective sport performance. Casting doubt on the straightforward application and interpretation of the primary findings, the primary effect-size prediction intervals, a more recent meta-analytic statistic reflecting the understanding of future results with comparable samples [16] encompassed 0. Moderation analyses helped pinpoint subgroups with no heterogeneity. All the results, whether significant or not, highlight ways to maximize the usefulness of the CSAI-2 or CSAI-2R with objective sport performance research.

### 4.1. Findings, Implications, and Clinical Significance

The results of the mean-difference analyses provide a perspective on how pre-competitive states separate successful and less successful performers. The perspective is cautionary, as the prediction intervals were wide, except in specific subgroups. Limitations of the studies themselves must contribute to the wide prediction intervals, along with the methodological differences amongst the included studies. With this caution in mind, the magnitude of these effects, particularly for self-confidence, suggests that successful athletes enter the competitive arena with a psychological profile characterized by higher self-belief and lower apprehensive arousal than their less successful counterparts. This group-level separation is most pronounced for the individual-sport win/loss subgroup, with prediction intervals ranging from no effects to exceptionally large effects for somatic anxiety and self-confidence [28,33,34,35,38,58]. These studies included only tennis and martial arts athletes. Importantly, the studies included male-only, female-only, and mixed samples; the CSAI-2 and CSAI-2R; and measurement the day of and within 60 min. Hence, there is more confidence that the results are related to tennis and martial art competitions than to other study variables. Two additional studies [57,61] provided win/loss data along with other comparison data for tennis and martial arts athletes; thus, we excluded those studies from the subgroup analyses. Of note, the effect-size values of the two excluded studies also support the reported subgroup effects.

A primary benefit of the mean-difference approach is its practical interpretability for coaches and consultants, especially with repeated measurements across events in line with Hanin’s original IZOF approach [72] and more recent advancements [73]. The win/loss effect sizes can convert into odds ratios. Though the conversion is an estimate, the interpretation is that the odds of winning are about “x” times higher for someone above the self-confidence median or below the cognitive anxiety and somatic anxiety medians (Borenstein, personal communication 19 June 2026, see Report S1 in the Supplemental Files for a conversion example). In our win/loss samples, the converted *g*-to-odds ratio prediction interval for self-confidence ranges from about 1 to 48; thus, the odds of winning are about none to 48 times higher for athletes scoring above the self-confidence median in a future study. In our win/loss data, tennis and martial artis athletes with higher self-confidence and lower somatic anxiety were more likely to perform better than those with lower self-confidence and higher somatic anxiety. Hence, within these two sports, even with heterogeneity, the CSAI-2 or 2R appears to have applied potential, certainly representing a line for future research to benefit the use of the CSAI in tennis and martial arts.

However, the better performer versus worse performer approach is not without limitations. First, with respect to the mean-difference results, especially for winning and losing, knowledge of the opponent’s ability and, thus, expectation of winning the match or the strength of the field must influence each athlete’s CSAI scores. Second, by categorizing athletes into groups such as winners versus losers or high versus low performers, we risk masking the individual variability that could contribute to the wide prediction intervals observed in this study as the mean-difference approach assumes a level of homogeneity within the successful group that may not exist. For instance, this approach overlooks athletes who performed well despite unconventional anxiety profiles (e.g., high cognitive anxiety utilized as facilitative arousal or higher somatic anxiety as facilitative excitement), highlighting the importance of Hanin’s IZOF approach [72], as well as the many self-regulation models [74] to best understand what an individual athletes’ CSAI scores mean in relation to the desired performance outcome. Lastly, we have no knowledge of what occurred from the moment of CSAI questionnaire completion to the event or thoughts during the event.

The group differences help quantify the CSAI scores that a practitioner must help an athlete bridge to reach a higher performance tier, while correlations provide information on the direction and strength of the findings. Potentially useful findings resulted from the included correlation-based studies. Our primary correlational data for cognitive anxiety and somatic anxiety reached statistical significance, providing evidence for the overall small detrimental impact of pre-competitive anxiety compared to data reported by Craft et al. [1] and Woodman and Hardy [2]. The primary self-confidence result aligns with values reported by Craft et al. [1], Woodman and Hardy [2], Lochbaum et al. [11], and Jekauc et al. [10] with different samples. The moderator results and specific subgroups provided stronger relational evidence, including no relationship present for placement, golf and gymnastics scores, and individual athlete statistics while performing on a team.

The three placement studies [20,25,43] in the analyses provided the largest mean correlation values for all three of the CSAI scales. However, two studies [20,43] provided large negative correlations for both anxieties, while Bois et al. provided non-supportive positive correlations for both anxieties. Hence, the placement results appear fragile, and placement is a rank; thus, Pearson correlation is not the appropriate statistic.

The results for golf and gymnastics scores and individual statistics on team sports provided data less fragile than placement, with instances with no heterogeneity. Studies on golf [36,46] and basketball [12,50,56] provided data across several competitive events, thereby strengthening the reported relationships, as opposed to one-event studies. Self-confidence when compared to both anxiety measures was most related to golf scores and individual basketball and volleyball game statistics. The clinical importance of relationships depends on the individual athlete, as well as the circumstances surrounding the athlete. For instance, at the highest level of golf, Rory Mcllroy’s season scoring average across the last 17 years of his career ranges from 68.32 to 70.35, with a standard deviation of 0.61. Nelly Korda’s scoring average across the last ten years ranges from 68.26 to 70.62, with a standard deviation of 0.74. Raising their self-confidence or lowering their anxieties by one standard-deviation unit, i.e., a Cohen’s *d* of 1.0, seems unattainable. However, at lower levels of competitive golf, where participants’ scores vary, self-regulation aimed at the CSAI constructs could be beneficial.

As for athlete statistics in a team sport, the results do support the use of the CSAI, especially for basketball, as a valuable pre-competition measure depending upon the athlete’s level. For instance, throughout his 23-year career, Lebron James’ points, rebounds, and assists calculated per 40 min of playing per game are incredibly consistent, as are Breanna Stewart’s field-goal and free-throw percentages across her 10-year WNBA career. Thus, with small standard deviations, any changes to their statistics (see Table S5) are a result of a myriad of game-specific variables such as shot selection, the defender, game importance, and nagging injuries mixed into pre-game confidence and anxieties. But again, with lower-level athletes or within specific situations, the CSAI constructs, as with the results, have a noticeable relationship with individual performances and, thus are of importance for the maintenance and improvement of performances across a season.

Lastly, measurement timing is an issue of historic and practical importance, with the recommendation of timing being within an hour of competition [1,3]. Though it is statistically true, with the adjusted *p*-value, that CSAI measurement within 1-hour emerged as the only significant timing effect size across both the mean-difference and correlation datasets, the prediction intervals were wide. Though not statistically significant, the day-of-measurement values were nearly identical to the 1-hour before correlations for cognitive and somatic anxiety. The day before competition yielded negligible sport performance relationships for both anxiety constructs. Surprisingly, this was not true for self-confidence. Thus, as the competitive moment approaches, the notion that cognitive states become more aligned with the impending performance is incorrect or the methodological differences are too great in the included studies to pinpoint the best time of measurement. On a positive note, greater flexibility in CSAI pre-competitive timing helps in the research process, given the longstanding issue of athlete accessibility being a barrier to consistent timing of CSAI measurement across and within studies.

### 4.2. Limitations and Future Directions

Limitations are inherent within the present and most, if not all, systematic sport psychology reviews and meta-analyses, with performance warranting consideration [7]. First, we restricted the search to peer-reviewed publications with the search conducted in English. While both are common approaches in sport psychology meta-analyses, this approach may have omitted pertinent gray literature and data published in non-English indexed journals. Our search strategy potentially limited the global representativeness of the findings, though countries from across the world are included in this meta-analysis, introducing bias. As noted by Lochbaum and Lane [7], sport psychology research is global; future syntheses would benefit from multilingual research teams capable of navigating non-English databases to provide a more culturally diverse picture of the pre-competition CSAI and performance literature or any literature (e.g., Lochbaum et al. [75] conducted searches in English and Turkish).

More substantive limitations include the limited number of similar study settings; the scarcity of within-team research; minimal measurement of historic CSAI ideas such as direction, facilitative or debilitative measurement, and testing for the curvilinear somatic anxiety–performance relationship; and testing of underlying or complementary physiological or psychosocial mechanisms or processes. In similar study settings, those involving tennis, martial arts, golf, and basketball provided insightful findings. Future studies could build on these results to further the CSAI and sport performance literature. The complex interplay of shared team dynamics and collective efficacy, which requires sophisticated multi-level modeling to resolve, could explain the negligible effect sizes observed in within-team studies. Consequently, we suggest consistent methodological shifts from one pre-performance CSAI measurement and one performance design toward intra-individual, longitudinal, and idiographic studies to better capture how fluctuations in psychological state map onto performance trajectories. Whether the shifts should account for perceptions of CSAI scale direction and Martens and colleagues’ original thoughts on the curvilinear relationship between somatic anxiety and sport performance are debatable. These ideas seem far from the reality of current CSAI studies.

Finally, while this review prioritizes the CSAI and objective sport performance, the potential psychosocial and physiological mechanisms underlying the CSAI states remain unknown. The integration of CSAI measurement with measures of psycho-biosocial experiences and biological markers is exemplified, in part, by Lu and colleagues’ [42] examination of sleep and mood. This type of research represents a pathway for future inquiry. Understanding the reciprocal relationship between pre-competitive anxiety, self-confidence, and other emotions or more detailed psycho-biosocial states and biomarkers like salivary cortisol or oxytocin will be essential for the development of a truly multidimensional understanding and prediction of objective sport performance.

## 5. Conclusions

The CSAI-2 has been prominent in sport psychology literature for over 40 years. Based on the included studies, researchers used the CSAI-2 as the preferred measure. However, researchers based on the included studies have increasingly used the CSAI-2R since 2023. The results provide a substantial update and specific instances of support for the use of the CSAI-2 or 2R in competitive sport. Practitioners should utilize the CSAI-2 or 2R the day of competition to help athletes identify their optimal zones, akin to Hanin’s IZOF. When following pre-competition anxieties and self-confidence, practitioners and future CSAI research must be prepared for and account for individual variability, as some athletes, based on the overall wide prediction intervals reported in the present meta-analyses, performed well despite high anxiety and low self-confidence and, conversely, perform poorly with low anxiety and high self-confidence.

## Figures and Tables

**Table 1:** Study characteristics.

Authors	N	Sport	CSAI	Performance	Data
Alejo et al. [18]	60 m	Boxing	2	Medalists, non-medalists	M
Alejo et al. [19]	58 m	Brazilian Jujutsu	2	Medalists, non-medalists	M
Aliberti et al. [20]	62 f	Dancesport	2R	Event ranking; high, low performers	r, M
Arroyo Del Bosque et al. [21]	95	Soccer	2	Winners, losers	M
Barnes et al. [22]	13 m	Swimming	2	Race time, intra-individual	r
Beattie et al. [23]	62	Slalom canoeing	2	Race time, intra-individual	r
Bejek & Hagtvet [24]	70 f	Gymnastics	2	Event score	r
Bois et al. [25]	41 m	Golf	2	Made cut, missed cut; final placement	r, M
Burton [26]	28; 70	Swimming	2	Race time, intra-individual	r
Caetano de Andrade Nogueira et al. [27]	15 m	Volleyball	2R	Won, lost (within same team)	M within
Chapman et al. [28]	142 m	Tae Kwon-Do	2	Winners, losers	M
Conde-Ripoll et al. [29]	28 m	Padel	2R	Winners, losers	M
Conde-Ripoll et al. [30]	10 m	Padel	2R	Winners, losers	M
Costa et al. [31]	16 m	Beach volleyball	2R	Winners, losers; event statistics	r, M
Dallas et al. [32]	68 m	Gymnastics	2	Higher, lower performers; event score	r, M
Fernandez-Fernandez et al. [33]	12 f	Tennis	2R	Winners, losers	M
Filaire et al. [34]	16	Tennis	2	Winners, losers	M
Fuentes-García et al. [35]	18 m	Tennis	2R	Winners, losers	M
Hassmen et al. [36]	8 m	Golf	2	Event score	r
Heazlewood & Burke [37]	416	Ironman	2	Race time	r
Jones et al. [4]	48 f	Gymnastics	2	Event score; good, poor performers	r, M
Kais & Raudsepp [38]	66 m	Beach volleyball	2	Event statistics	r
Kenshloo et al. [39]	30 f	Karate	2R	Winners, losers	r (M)
Krane & Williams [40]	216	Track and field	2	Placers, non-placers	M
La Fratta et al. [41]	56 m	Soccer	2	Winners, losers	M
León-Prados et al. [12]	9 m; 9 f	Basketball	2	Event statistics	r
Lu et al. [42]	23	Shooting	2	Event score	r
Mabweazaa et al. [43]	61 m	Swimming	2	Event place	r
Martin & Gill [44]	73 m	Track and field	2	Race time; event place	r
Mateo-Orcajada et al. [45]	5 m	Esports	2	Won, lost (within team)	M within
McAuley [46]	7 f	Golf	2	Event score	r
McCann et al. [47]	23	Cycling	2	Race time	r
Mon-Lopez et al. [48]	23 f	Shooting	2	Event score	r
Papastergiou et al. [49]	264	Handball	2	Winners, losers	M
Parfitt & Pates [50]	12 m	Basketball	2	Event statistics	r
Pinto & Vázquez [51]	77	Golf	2	Event score	r
Raudsepp & Kais [52]	54 m	Beach volleyball	2	Event statistics	r
Sanchez et al. [53]	19 m	Climbing	2	Event score; successful, unsuccessful	r, M
Silva et al. [54]	35	Swimming	2	Superior, inferior performance	M
Slačanac et al. [55]	200 m	Wrestling	2	Placement	r
Swain & Jones [56]	10 m	Basketball	2	Event statistics	r
Terry et al. [57]	100	Tennis	2	Winners, losers (singles, doubles)	M
Terry & Slade [58]	104 m	Martial arts	2	Winners, losers	M
Tsopani et al. [59]	86 f	Rhythmic gymnastics	2	High, low scorers; finalists, non-finalists; event score	r, M
Van Der Heever et al. [60]	144 f	Netball	2	Successful, unsuccessful	M
Vlachopoulos et al. [61]	402	Martial arts	2R	Winners, losers; good, poor performers	M
Williams & Krane [62]	83 f	Golf	2	Event score	r
Zhang et al., 2024 [63]	106	Table tennis	2R	Event statistics	r

Abbreviations: m = all-male sample; f = all-female sample; nothing listed = mixed sample; 2 = CSAI-2 version; 2R = CSAI-2R version; M = contributed mean data; r = contributed correlational data.

**Table 2:** Primary results for the mean difference between group samples.

Group	*k*	*g*	*n*	95% CI	*p*-Value	PI	Q	Tau^2^	*I* ^2^	FS	Orwin	Trim–Fill
CA	24	−0.52	1993	−0.78, −0.26	<0.01	−1.73, 0.69	152.48	0.32	84.92	849	40	3L
SA	24	−0.37	1993	−0.61, −0.13	<0.01	−1.44, 0.71	128.34	0.25	82.08	440	21	3L
SC	25	0.59	2049	0.36, 0.82	<0.01	−0.48, 1.66	129.07	0.25	81.40	1192	48	0

Abbreviations: CA = cognitive anxiety; SA = somatic anxiety; SC = self-confidence; *k* = samples; CI = confidence interval; PI = prediction interval. L = data added to the left of the mean.

**Table 3:** Moderation results with subgroupings for the mean-difference samples.

CSAI Scale	Grouping	*k*	*n*	*g*	95% CI	*p*-Value	PI
CA	Individual result	5	455	−0.38	−1.04, 0.28	0.26	−2.85, 2.09
	Researcher-defined	6	373	−0.41	−0.94, 0.13	0.13	−1.76, 0.95
	Win/loss—individual	6	417	−0.95	−1.52, −0.37	<0.01	−2.32, 0.43
	Win/loss—team	5	376	−0.40	−0.99, 0.19	0.18	−1.73, 0.69
SA	Individual result	4	381	−0.38	−0.93, 0.16	0.17	−1.54, 0.78
	Researcher-defined	6	373	−0.10	−0.56, 0.36	0.67	−1.22, 1.02
	Win/loss—individual	6	417	−1.06	−1.57, −0.55	<0.01	−2.21, 0.09
	Win/loss—team	5	376	−0.15	−0.66, 0.36	0.56	−1.29, 0.99
CON	Individual result	4	381	0.05	−0.46, 0.56	0.84	−1.04, 1.14
	Researcher-defined	6	373	0.61	0.17, 1.05	<0.01	−0.44, 1.67
	Win/loss—individual	6	417	1.06	0.57, 1.54	<0.01	−0.02, 2.13
	Win/loss—team	6	432	0.41	−0.03, 0.86	0.07	−0.64, 1.47
CA	<=60 min	13	1286	−0.67	−1.01, −0.32	<0.01	−1.88, 0.55
	Day of	7	353	−0.56	−1.05, −0.06	0.03	−1.83, 0.72
	Day before	4	354	−0.00	−0.59, 0.59	1.00	−1.32, 1.32
SA	<=60 min	13	1286	−0.44	−0.77, −0.12	<0.01	−1.56, 0.67
	Day of	7	353	−0.56	−1.03, −0.09	0.02	−1.73, 0.61
	Day before	4	354	−0.09	−0.45, 0.63	0.75	−1.12, 1.31
SC	<=60 min	13	1286	0.70	0.37, 1.03	<0.01	−0.45, 1.85
	Day of	7	353	0.37	−0.11, 0.84	0.13	−0.83, 1.57
	Day before	5	410	0.61	0.09, 1.12	0.02	−0.61, 1.83

Abbreviations: CA = cognitive anxiety; SA = somatic anxiety; SC = self-confidence; *k* = samples; *n* = total participants in group; CI = confidence interval; PI = prediction interval.

**Table 4:** Primary results for the correlation samples.

Group	*k*	*r*	*n*	95% CI	*p*-Value	PI	Q	Tau^2^	*I* ^2^	FS	Orwin	Trim–Fill
CA	29	−0.18	1708	−0.29, −0.05	<0.01	−0.64, 0.39	141.26	0.08	80.18	340	14	0
SA	22	−0.24	1278	−0.39, −0.08	<0.01	−0.75, 0.44	136.25	0.11	84.59	414	36	4L
SC	25	0.26	1576	0.12, 0.39	<0.01	−0.37, 0.72	147.17	0.09	83.69	481	9	0

Abbreviations: CA = cognitive anxiety, SA = somatic anxiety, SC = self-confidence, *k* = samples, CI = confidence interval, PI = prediction interval. L = data added to the left of the mean.

**Table 5:** Moderation and selected subgroup results for the correlation samples.

CSAI Scale	Groupings	*k*	*n*	*r*	95% CI	*p*-Value	PI
CA	IND Sports—All Measures	21	1426	−0.16	−0.30, −0.02	0.02	−0.65, 0.42
	Placement	3	173	−0.54	−0.72, −0.28	<0.01	−0.83, −0.00
	All Scores	11	511	−0.04	−0.21, 0.15	0.71	−0.51, 0.46
	Golf Score	4	175	−0.19	−0.34, −0.04	=0.01	95% CI is PI
	Gymnastics Score	4	271	0.02	−0.11, 0.14	0.80	95% CI is PI
	Race Time	4	593	−0.00	−0.21, 0.20	0.98	−0.48, 0.47
	Race Time Adjusted to Individual	3	149	−0.30	−0.54, −0.02	0.03	−0.71, 0.26
	IND Statistics on a Team	7	176	−0.14	−0.29, 0.02	0.09	95% CI is PI
	Basketball Statistics	4	40	−0.37	−0.64, −0.02	0.04	95% CI is PI
	Volleyball Statistics	3	136	−0.08	−0.25, 0.09	0.36	95% CI is PI
SA	IND Sports—All Measures	17	1120	−0.27	−0.43, −0.09	<0.01	−0.77, 0.44
	Placement	3	173	−0.53	−0.77, −0.17	<0.01	−2.76, 0.01
	All Scores	9	359	−0.12	−0.37, 0.14	0.37	−0.89, 0.37
	Golf Score	4	133	0.03	−0.19, 0.24	0.77	−0.34, 0.40
	Gymnastics Score	3	202	−0.18	−0.18, 0.18	0.97	−0.33, 0.34
	Race Time	2	439	−0.37	−0.52, −0.20	<0.01	−0.66, 0.02
	Race Time Adjusted to Individual	3	149	−0.12	−0.32, 0.09	0.27	−0.52, 0.32
	IND Statistics on a Team	5	158	−0.12	−0.43, −0.09	0.52	−0.80, 0.44
	Basketball Statistics	2	22	−0.20	−0.60, 0.28	0.42	95% CI is PI
	Volleyball Statistics	3	136	−0.10	−0.26, 0.08	0.28	95% CI is PI
SC	IND Sports—All Measures	19	1409	0.21	0.06, 0.35	<0.01	−0.38, 0.68
	Placement	3	312	0.44	0.18, 0.64	<0.01	−0.08, 0.77
	All Scores	10	428	0.19	0.01, 0.36	0.04	−0.30, 0.60
	Golf Score	3	92	0.34	0.09, 0.54	<0.01	−0.04, 0.63
	Gymnastics Score	4	271	0.20	0.06, 0.33	<0.01	−0.08, 0.45
	Race Time	3	520	−0.02	−0.31, 0.26	0.87	−0.52, 0.49
	Race Time Adjusted to Individual	3	149	0.20	−0.13, 0.50	0.23	−0.36, 0.66
	IND Statistics on a Team	6	167	0.45	0.17, 0.66	<0.01	−0.19, 0.82
	Basketball Statistics	3	92	0.56	0.18, 0.79	<0.01	−0.08, 0.87
	Volleyball Statistics	3	271	0.43	0.22, 0.60	<0.01	−0.01, 0.73
CA	<=60 min	17	631	−0.31	−0.46, −0.14	<0.01	−0.74, 0.29
	Day of	7	434	−0.09	−0.31, 0.32	0.49	−0.62, 0.51
	Day before	5	643	0.06	−0.22, 0.16	0.69	−0.54, 0.61
SA	<=60 min	13	459	−0.33	−0.53, −0.10	<0.01	−0.82, 0.44
	Day of	5	176	−0.08	−0.45, 0.31	0.69	−0.75, 0.67
	Day before	4	643	−0.16	−0.47, 0.18	0.37	−0.77, 0.60
SC	<=60 min	14	488	0.37	0.19, 0.53	<0.01	−0.24, 0.77
	Day of	6	445	0.15	−0.11, 0.39	0.27	−0.47, 0.67
	Day before	5	643	0.16	−0.12, 0.41	0.27	−0.47, 0.68

Abbreviations: CA = cognitive anxiety; SA = somatic anxiety; SC = self-confidence; *k* = samples; CI = confidence interval; PI = prediction interval.

## Data Availability

All data are contained in the manuscript and Supplemental Files.

## Supplementary Materials

The following supporting information can be downloaded at: https://www.mdpi.com/article/10.3390/sports14080334/s1, Table S1: Definitions of statistics and tests reported in the meta-analysis as defined within the Comprehensive Meta-Analysis program; Table S2: Hoy and colleagues’ [69] risk of bias tool; Table S3. Study characteristics beyond information found in Table 1; Table S4: Risk of bias within studies ratings; Figure S1: Distribution of true effects for mean difference (*g*) samples. Cognitive anxiety (top), somatic anxiety (middle), and confidence (bottom); Figure S2: Figures associated with Caetano de Andrade Nogueira et al. [27] and Mateo-Orcajada et al. [45]; Figure S3: One-study removed for mean difference (*g*) samples. Cognitive anxiety studies [4,18,19,20,21,25,27,28,29,30,31,32,33,34,35,39,40,49,53,54,58,59,60,61] (top figure), somatic anxiety studies [4,18,19,20,21,25,27,28,29,30,31,32,33,34,35,39,40,49,53,54,58,59,60,61] (middle figure), and self-confidence studies, in order [4,18,19,20,21,25,27,28,29,30,31,32,33,34,35,39,40,41,49,53,54,58,59,60,61] (bottom figure); Figure S4: Cumulative analysis by year for mean difference (*g*) samples. Cognitive anxiety studies [4,18,19,20,21,25,27,28,29,30,31,32,33,34,35,39,40,49,53,54,58,59,60,61] (top figure), somatic anxiety studies [4,18,19,20,21,25,27,28,29,30,31,32,33,34,35,39,40,49,53,54,58,59,60,61] (middle figure), and self-confidence studies, in order [4,18,19,20,21,25,27,28,29,30,31,32,33,34,35,39,40,41,49,53,54,58,59,60,61] (bottom figure); Figure S5: Distribution of true effects for mean difference (*r*) samples. Cognitive anxiety (top), somatic anxiety (middle), and confidence (bottom); Figure S6: One-study removed for mean difference (*r*) samples. Cognitive anxiety studies, in order [12,20,22,23,24,25,26,31,32,36,37,38,42,43,44,46,47,48,50,51,52,53,56,59,62,63] (top figure), somatic anxiety studies, in order [20,22,25,26,31,32,36,37,38,42,43,46,47,48,50,51,52,53,56,59] (middle figure), and self-confidence studies [4,12,20,22,23,24,25,26,31,32,38,42,46,47,48,50,51,52,53,55,56,59]; Figure S7: Cumulative analysis by year for mean difference (*r*) samples. Cognitive anxiety studies, in order [12,20,22,23,24,25,26,31,32,36,37,38,42,43,44,46,47,48,50,51,52,53,56,59,62,63] (top figure), somatic anxiety studies, in order [20,22,25,26,31,32,36,37,38,42,43,46,47,48,50,51,52,53,56,59] (middle figure), and self-confidence studies [4,12,20,22,23,24,25,26,31,32,38,42,46,47,48,50,51,52,53,55,56,59]. Report R1: Effect size, odds ratio conversion, and interpretation: Self-confidence winners vs. losers. Table S5: L. James and B. Steward data sets.

## Abbreviations

The following abbreviations are used in this manuscript:

CSAI-2	Competitive State Anxiety Inventory-2
CSAI-2R	Revised Competitive State Anxiety Inventory-2
CA	Cognitive anxiety
SA	Somatic anxiety
CON	Self-confidence

## Appendix A

### Appendix A.1

**Table A1:** The 27-item CSAI-2. Adapted from Martens et al. [3]. Items 3, 6, 12, 14, and 21 were reverse scored before summing. Subscale scores range from 9 to 36.

Item Number	Question/Statement	Subscale
1	I am concerned about this competition.	Cognitive Anxiety
2	I feel nervous.	Somatic Anxiety
3	I feel at ease.	Self-Confidence
4	I have self-doubts.	Cognitive Anxiety
5	I feel jittery.	Somatic Anxiety
6	I feel comfortable.	Self-Confidence
7	I am concerned I may not do as well in this competition as I could.	Cognitive Anxiety
8	My body feels tense.	Somatic Anxiety
9	I feel self-confident.	Self-Confidence
10	I am concerned about losing.	Cognitive Anxiety
11	I feel tense in my stomach.	Somatic Anxiety
12	I feel secure.	Self-Confidence
13	I am concerned about choking under pressure.	Cognitive Anxiety
14	My body feels relaxed.	Somatic Anxiety
15	I’m confident I can meet the challenge.	Self-Confidence
16	I am concerned about performing poorly.	Cognitive Anxiety
17	My heart is racing.	Somatic Anxiety
18	I’m confident about performing well.	Self-Confidence
19	I’m concerned about reaching my goal.	Cognitive Anxiety
20	I feel my stomach sinking.	Somatic Anxiety
21	I feel mentally relaxed.	Self-Confidence
22	I’m concerned that others will be disappointed with my performance.	Cognitive Anxiety
23	My hands are clammy.	Somatic Anxiety
24	I’m confident because I mentally picture myself reaching my goal.	Self-Confidence
25	I’m concerned I won’t be able to concentrate.	Cognitive Anxiety
26	My body feels tight.	Somatic Anxiety
27	I’m confident of coming through under pressure.	Self-Confidence

### Appendix A.2

**Table A2:** The 17-item CSAI-2R. Adapted from Cox et al. [6]. Respondents rate each item on a 4-point Likert scale: 1 (not at all), 2 (somewhat), 3 (moderately so), or 4 (very much so).

Item Number	Question/Statement	Subscale
1	I feel jittery.	Somatic Anxiety
2	I am concerned about this competition.	Cognitive Anxiety
3	I feel self-confident.	Self-Confidence
4	My body feels tense.	Somatic Anxiety
5	I am concerned about losing.	Cognitive Anxiety
6	I feel tense in my stomach.	Somatic Anxiety
7	I’m confident I can meet the challenge.	Self-Confidence
8	I am concerned about choking under pressure.	Cognitive Anxiety
9	My heart is racing.	Somatic Anxiety
10	I’m confident about performing well.	Self-Confidence
11	I’m concerned about performing poorly.	Cognitive Anxiety
12	I feel my stomach sinking.	Somatic Anxiety
13	I’m confident because I mentally picture myself reaching my goal.	Self-Confidence
14	I’m concerned that others will be disappointed with my performance.	Cognitive Anxiety
15	My hands are clammy.	Somatic Anxiety
16	I’m confident of coming through under pressure.	Self-Confidence
17	My body feels tight.	Somatic Anxiety
