# Supplementary material for: A Meta-Analysis of the CSAI-2 and CSAI-2R with Objective Sport Performance from 1985–2026
Source: Sports (Basel). 2026 Aug 4;14(8):334. doi: 10.3390/sports14080334 (PMC13517692; doi:10.3390/sports14080334)
Supplement: Supplementary file 1 [file sports-14-00334-s001.zip › sports-4336632-supplementary.pdf]

Supplemental Figures and Tables

- **Table S1.** Definitions of statistics and tests reported in the meta-analysis as defined within the Comprehensive Meta-Analysis program.
- **Table S2.** Hoy and colleagues' [69] risk of bias tool.
- **Table S3.** Study characteristics beyond information found in Table 1.
- **Table S4.** Risk of bias within studies ratings.
- **Figure S1**. Distribution of true effects for mean difference (g) samples. Cognitive anxiety (top), somatic anxiety (middle), and confidence (bottom).
- **Figure S2**. Figures associated with Caetano de Andrade Nogueira et al. [27] and Mateo-Orcajada et al. [45].
- **Figure S3.** One-study removed for mean difference (*g*) samples. Cognitive anxiety studies [18-21,25,27-35,4,39,40,49,53,54,58,59,60,61] (top figure), somatic anxiety studies [18-21,25,27-35,4,39,40,49,53,54,58-61] (middle figure), and self-confidence studies [in order 18-21,25,27-35,4,39-41,49,53,54,58-61] (bottom figure).
- **Figure S4**. Cumulative analysis by year for mean difference (*g*) samples. Cognitive anxiety studies [18-21,25,27-35,4,39,40,49,53,54,58,59,60,61] (top figure), somatic anxiety studies [18-21,25,27-35,4,39,40,49,53,54,58-61] (middle figure), and self-confidence studies [in order 18-21,25,27-35,4,39-41,49,53,54,58-61] (bottom figure).
- **Figure S5.** Distribution of true effects for mean difference (*r*) samples. Cognitive anxiety (top), somatic anxiety (middle), and confidence (bottom).
- **Figure S6.** One-study removed for mean difference (*r*) samples. Cognitive anxiety studies [in order 20,22-26,31,32,36-38,12,42-44,46-48,50-53,56,59,62,63] (top figure), somatic anxiety studies [in order 20,22,25,26,31,32,36-38,42,43,46-48,50-53,56,59] (middle figure), and self-confidence studies [20,22-26,31,32,4,38,12,42,46-48,50-53,55,56,59].
- **Figure S7.** Cumulative analysis by year for mean difference (*r*) samples. Cognitive anxiety studies [in order 20,22-26,31,32,36-38,12,42-44,46-48,50-53,56,59,62,63] (top figure), somatic anxiety studies [in order 20,22,25,26,31,32,36-38,42,43,46-48,50-53,56,59] (middle figure), and self-confidence studies [20,22-26,31,32,4,38,12,42,46-48,50-53,55,56,59].

- **Report S1.** Effect size, odds ratio conversion, and interpretation: Self-confidence winners vs. losers

Table S1. Definitions of statistics and tests reported in the meta-analysis as defined within the Comprehensive Meta-Analysis program.

**Risk of Bias Statistic:** Definition
**Random-effects model**: This model applies when there is a universe of populations that we care about and we can assume that the studies in the analysis are a random sample of those populations. The results of our analysis will be generalized to that universe. In general, when the studies in the analysis are pulled from the literature, this is the model we should be using. This is the model that allows us to generalize to the universe of comparable studies, which is typically what we intend to do. At the same time, we need to be aware of the limitations of this model when employed in this way. For the model to work properly we need a sufficient number of studies to yield a reliable estimate of tau-squared, the between-study variance. Additionally, while we can say that the results apply to the universe of comparable studies, it may not be clear what that universe includes.
**The Q-test for heterogeneity**: The Q-test is intended to address the question "Is there evidence that the true effect size varies (at all) across studies". The criterion alpha for the Q-test is typically set at 0.100. The rationale for this is that the test often has low statistical power, and that using a criterion of 0.100 makes it more likely that we will be able to reject the null hypothesis and prove that the effect size varies across studies. This test answers the following question: Is there evidence of ANY variation in effect size?
**The I-squared statistic**: There is a common belief that I-squared tells us how much the effect size varies across studies. In some cases, I-squared values have been employed to classify the amount of variation as being low, moderate, or high. While this interpretation of I-squared is ubiquitous in some fields, it is nevertheless incorrect. I-squared is a proportion, not an absolute amount. It tells us what proportion of the variance in observed effects reflects variance in true effects rather than sampling error. It does not tell us how much the effects actually vary. The statistic that tells us how much the effect size varies is the prediction interval. This statistic answers the following question: Of the variance that we see in the observed effects, what proportion reflects variation in true effects rather than sampling error?
**Tau-squared**: The variance of true effect sizes. This statistic answers the following question: What is the variance of true effects?
**The prediction interval**: One function of the prediction interval is to provide context for understanding the mean effect size. If the prediction interval tells us that the effect size is consistent across studies, the impact of the intervention will be similar for all relevant populations, and it would probably be useful to focus on the mean effect size. By contrast, if the prediction interval tells us that the effect size varies substantially across populations, the mean becomes less useful as a summary statistic. Rather, we would want to understand the range of effects. The prediction interval may allow us to report, that (a) the effect size is always helpful, but varies from a trivial to a moderate effect, or (b) the effect size is always helpful, and varies from a moderate to a large effect, or (c) the effect is helpful in some cases but

may be harmful in others. The accuracy of the prediction interval depends on several assumptions, including the assumption that the effects are normally distributed about the mean. Therefore, if the prediction interval includes effects that indicate an intervention is harmful it would be important to see if there are actually observed effects that support this conclusion. This statistic answers the following question: In what interval do the true effects fall?
**Orwin's Fail-Safe *n***: This metric estimates the number of undetected studies required to shift the observed correlation below a specific meaningful threshold. For this analysis, the missed study value was set to zero, and a threshold of ± 0.10 was used to represent the minimum bound for a small effect size.
**Classic Fail-Safe *n***: This calculation determines the number of null-result studies needed to render a statistically significant effect non-significant. A one-tailed test was applied for this analysis.
**Interpretation of *n* values**: Higher values for both *n* calculations indicate greater robustness and increased confidence that the findings are not significantly undermined by publication bias.
**Funnel Plot Visualization**: Funnel plots were visually inspected to assess whether included studies were distributed symmetrically around the mean effect size, which suggests that the available literature is representative of the entire population of relevant studies.
**Duval and Tweedie's Trim and Fill Method**: This method was applied to account for potential study suppression by identifying and imputing missing data points. Data points added to the right side of the plot inflate the cumulative effect size, while those added to the left decrease it.

Table S2. Adapted version of Hoy and colleagues' (2012) risk of bias tool.

**Item**	**Low Risk**	**Medium Risk**	**High Risk**
1 - was the study's target population a close representation of the national population in relation to relevant variables?	Study purpose states a specific target population such as elite tennis players and the participants are the target population.	The study included some participants in other sports.	Study purpose states a specific target population but the sample is a mix of sports within the same data set.
2 - was the sample frame a true or close representation of the target population?	Participant level clearly indicated providing confidence that the frame is a true or close representation of the target population	The study seemed to include participants in other sports or ability levels to recruit enough participants.	Study purpose states a specific target population but the sample is a mix of sports within the same data set.
3 - was some form of random selection used to select the sample OR was a census undertaken?	Random selection of some form used.	Random selection from within a team.	No random selection of any kind.
4 - was the likelihood of nonresponse bias minimal?	Studies with < 100 participants needed to achieve 80% rate.<br><br>Studies > 100 participants.	Reports of non-response that seemed < 80% for groups that are small OR team data collection without details of how many team members there were.	Data collected at a large event whereby the researchers recruited for participation, and they did not recruit at least 100 participants.
5 - were the data collected directly from the subjects (as opposed to a proxy)? The 'a proxy' does not apply to us so just the first part - collected directly.<br><br>Added - collected directly from the subjects by the research team	Yes, procedures stated specifically without question collected directly from the subjects by the research team.	Most likely by the research team.	Not in person such as mailed and returned or even electronically OR by coaching staff.

as if by anyone else, we do not know if subject A was subject A.			
6 - I am thinking this is for the performance measure.	Performance outcomes that are based on only the athlete such as intra-individual race time (e.g., Burton); golf and gymnastic scores	Performance outcomes that are based partially on performance of others such as Individual statistics within a team setting; individual outcome such as medaled, finalist, individual placement	Performances based on a lot of others such team win, lose, etc.; as well as vague mean difference outcomes - good, bad performance
7 -was the study instrument valid and reliable?	CSAI-2R - all 17 items	CSAI-2 - all 27 items or the Greek version made with 15 items, or minimal modification	Extremely modified version
8 - was the same mode of data collection used for all participants?	Yes, same mode for all participants.	Unsure, or appeared perhaps self-regulated by participants but even this is the same mode.	No, a mix of modes such as coach collected some, research staff collected some of the CSAI data.
9 - was the length of the parameter of interest appropriate?	= < 60 min before the athlete's performance	Day off regardless of time as from CSAI completion to end of each athlete's event could be a long time	Day before
10 - were the numerator(s) and denominator(s) for the parameter of interest appropriate?	Multiple CSAI and multiple performance measures	One CSAI and one performance measure	One CSAI with multiple performances
11 - Summary score			

Table S3. Study characteristics beyond information found in Table 1.

Authors	Context	Participant Description	Athlete Level
Aliberti et al., 2024	Local competition	B, C, and D class	Recreational
Armas Alejo et al., 2025	Brazilian Jujutsu World Championships	All levels - white to black belt	Mix
Armas Alejo et al., 2020	Brazilian National Championship	Federated athletes - adult, young adult divisions	Advanced
Arroyo Del Bosque et al., 2022	League Championships of Vitoria-Gasteiz	Club, youth development program	Youth
Barnes et al., 1986	Big 8 Men's Swimming Championships	University	Advanced
Beattie et al., 2004	Eight competitive season events	British premier, 1st, 2nd divisions	Mix
Bejek & Hagtvet, 1996	Hungarian Junior National Championships	Hungarian National Junior Women's Team members and non-members	Advanced; youth
Bois et al., 2009	The Open of Bordeaux	Professional	Elite
Burton, 1988	Meets and Big 10 Championship; 1982 National Sports Festival in Indianapolis	University; Selected for Festival	Advanced; Intermediate
Caetano de Andrade Nogueira et al., 2024	24 competitive season matches	U18 category of a professional elite volleyball team	Elite
Chapman et al., 1997	1996 British University Student Tae Kwon-Do Championships	University	Advanced
Conde-Ripoll et al., 2023	2023 Finnish Padel Championship	Ranked top 103 in Finland	Elite

Conde-Ripoll et al., 2024	2023 Finnish National Championship by pairs	Ranked top 25 in Finland	Elite
Costa et al., 2019	Researcher organized 1 match per team event with a prize	National circuit competitors, some with World Championship experience	Elite
Dallas et al., 2019	Greek National competition	Clubs affiliated with the Greek Gymnastics Federation	Youth
Fernandez-Fernandez et al., 2014	U-14 tennis competition on the national and international level	Elite youth with national ranking between 5-20	Elite
Filaire et al., 2009	Regional tournament	Regional tennis players	Intermediate
Fuentes-García et al., 2023	2021 Borotra Cup Tennis Europe U16 Tournament	Elite U16	Advanced
Hassmen et al., 2004	10 most important events of Swedish National Amateur golf team's season	Swedish National Amateur Golf Team	Advanced
Heazlewood & Burke, 2011	Ironman Triathlon	Trained at least prior 10 months to Ironman	Mixed
Jones et al., 1993	Researcher organized local beam club competition	Club athletes from south of England	Intermediate
Kais & Raudsepp, 2004	Estonian National Championships, International World Tour, or European Championships	Competitive at Estonian national or international level	Elite
Kenshloo et al., 2026	Internal selection for women's national team competition	Elite athletes recruited from Iranian national team camp	Elite
Krane & Williams, 1994	Prestigious invitational relay track and field meet	University and high schoolers having met high standards to enter event	Advanced; Intermediate

La Fratta et al., 2021	Competitive season tournament	Youth players of River '65 Elite Soccer School	Advanced
León-Prados et al., 2014	6 games; 4 games	1st Division National Women (Liga Femenina); provincial youth	Elite; Youth
Lu et al., 2022	National shooting competition	Elite Shanghai air-rifle shooting team	Elite
Mabweazaa et al., 2016	Inter-school swimming gala in Zimbabwe	Private high school in Zimbabwe, 4+ years of swimming experience	Intermediate
Martin & Gill, 1991	Midseason dual track meet	High school distance runners	Intermediate
Mateo-Orcajada et al., 2022	18 matches in the SuperLiga Orange league	Professional League of Legend staters from same team	Elite
McAuley, 1985	10 Inter-collegiate golf events	University of Iowa women's golf team	Advanced
McCann et al., 1992	Senior National Developmental Camps at US Olympic Training Center	Elite seniors	Elite
Mon-Lopez et al., 2022	Spanish Olympic Shooting Championship	Minimum of 500 points in previous competitions	Elite
Papastergiou et al., 2010	Greek National Handball Championship	National youth	Advanced
Parfitt & Pates, 1999	3-4 games in Welsh, British Inter-University, and Polytechnic Leagues or Birmingham Premier League	Mix of university and Birmingham Premier League players	Advanced, elite mix
Pinto & Vázquez, 2013	National Argentina 2010 Golf Tournament	Amateurs <18 years of age	Mix
Raudsepp & Kais, 2002	Estonian National, World or	Competitive at Estonian national or	Elite

	European Championship	international level	
Sanchez et al., 2010	Finals of the Belgian Climbing Championship	Elite	Elite
Silva et al., 2023	Brazilian Youth Swimming Championship	3+ years of participation in municipal and state-level competitions	Intermediate
Slačanac et al., 2021	Croatian National Greco-Roman Style Championships	National level cadets and juniors	Advanced
Swain & Jones, 1996	6 games in the Leicestershire Senior Division One League	Loughborough University men's team	Advanced
Terry, Cox, et al., 1996	1994 National Junior Championships, the Lawn Tennis Association Winter Series, or the Senior County Week tournament	At least county standard	Advanced
Terry & Slade, 1995	Regional competitions	Brown or black belt status	Mix
Tsopani et al., 2011	Greek National Competition (final phase)	Club athletes associated with Greek Gymnastics Federation	Mix
Van Der Heever et al., 2007	South African Inter-Provincial Netball Tournaments	Junior provincial athletes	Intermediate
Vlachopoulos et al., 2025	Described only as competitions	Club, national, and international level	Mix
Williams & Krane, 1992	Prestigious Southwestern Collegiate Invitational Golf Tournament	From 18 DI women's golf teams	Advanced
Zhang et al., 2024	Chinese national table tennis championship	Elite	Elite

Table S4. Risk of bias within studies ratings.

Authors	1	2	3	4	5	6	7	8	9	10
Aliberti et al., 2024	Low risk	Low risk	High risk	High risk	Medium risk	Medium risk; High risk	Low risk	Low risk	Low risk	Medium risk
Armas Alejo et al., 2025	Low risk	Low risk	High risk	High risk	Low risk	Medium risk	Medium risk	Low risk	Medium risk	High risk
Armas Alejo et al., 2020	Low risk	Low risk	High risk	High risk	Low risk	Medium risk	Medium risk	Low risk	High risk	High risk
Arroyo Del Bosque et al., 2022	Low risk	Low risk	High risk	High risk	Medium risk	Medium risk	Medium risk	Low risk	Low risk	Medium risk
Barnes et al., 1986	Low risk	Low risk	High risk	Medium risk	Medium risk	Low risk	High risk	Low risk	Low risk	Medium risk
Beattie et al., 2004	Low risk	Low risk	High risk	Medium risk	Medium risk	Low risk	Medium risk	Low risk	Low risk	Low risk
Bejek & Hagtvet, 1996	Low risk	Low risk	High risk	High risk	Medium risk	Low risk	Medium risk	Low risk	Medium risk	High risk
Bois et al., 2009	Low risk	Low risk	High risk	High risk	Medium risk	Medium risk; Medium risk	Medium risk	Medium risk	High risk	High risk
Burton, 1988	Low risk	Low risk	High risk	Medium risk;	Medium risk	Low risk	Medium risk	Low risk	Low risk	Medium risk;

				Medium risk						Medium risk
Caetano de Andrade Nogueira et al., 2024	Low risk	Low risk	High risk	Low risk	Medium risk	High risk	Low risk	Low risk	Low risk	Low risk
Chapman et al., 1997	Low risk	Low risk	High risk	Low risk	Medium risk	High risk	Medium risk	Low risk	Medium risk	High risk
Conde-Ripoll et al., 2023	Low risk	Low risk	High risk	Low risk	Medium risk	High risk	Low risk	Low risk	Low risk	Low risk
Conde-Ripoll et al., 2024	Low risk	Low risk	High risk	Medium risk	Medium risk	High risk	Low risk	Low risk	Low risk	Low risk
Costa et al., 2019	Low risk	Low risk	High risk	Low risk	Medium risk	High risk; Medium risk	Low risk	Low risk	Low risk	Medium risk
Dallas et al., 2019	Low risk	Low risk	High risk	Low risk	Low risk	High risk; Low risk	Medium risk	Low risk	Medium risk	High risk
Fernandez-Fernandez et al., 2014	Low risk	Low risk	High risk	High risk	Medium risk	High risk	Low risk	Low risk	Low risk	Medium risk
Filaire et al., 2009	Low risk	Low risk	High risk	High risk	Medium risk	High risk	Medium risk	Low risk	Medium risk	Medium risk
Fuentes-García et al., 2023	Low risk	Low risk	High risk	High risk	Medium risk	High risk	Low risk	Low risk	Medium risk	Medium risk

Hassmen et al., 2004	Low risk	Low risk	High risk	Low risk	High risk	Low risk	Medium risk	Medium risk	Low risk	Low risk
Heazlewood & Burke, 2011	Low risk	Low risk	High risk	Low risk	High risk	Low risk	Medium risk	Low risk	High risk	Medium risk
Jones et al., 1993	Low risk	Low risk	High risk	Medium risk	Medium risk	Low risk; High risk	Medium risk	Low risk	Low risk	Medium risk
Kais & Raudsepp, 2004	Low risk	Low risk	High risk	High risk	Medium risk	Medium risk	Medium risk	Low risk	Medium risk	High risk
Kenshloo et al., 2026	Low risk	Low risk	High risk	Medium risk	Medium risk	High risk	Medium risk	Medium risk	Medium risk	High risk
Krane & Williams, 1994	Low risk	Low risk	High risk	Low risk	High risk	Medium risk	Medium risk	Low risk	Low risk	Low risk
La Fratta et al., 2021	Low risk	Low risk	High risk	Low risk	Medium risk	High risk	Medium risk	Low risk	High risk	Medium risk
León-Prados et al., 2014	Low risk	Low risk	High risk	Low risk	Medium risk	Medium risk	Medium risk	Low risk	Low risk	Low risk
Lu et al., 2022	Low risk	Low risk	High risk	Low risk	Medium risk	Low risk	Medium risk	Low risk	High risk	Medium risk
Mabweazaa et al., 2016	Low risk	Low risk	High risk	Medium risk	Medium risk	Medium risk	Medium risk	Low risk	Low risk	Medium risk
Martin & Gill, 1991	Low risk	Low risk	High risk	Medium risk	Medium risk	Low risk; Medium risk	Medium risk	Low risk	Low risk	Medium risk

Mateo-Orcajada et al., 2022	Low risk	Low risk	High risk	Low risk	High risk	High risk	Medium risk	Low risk	Low risk	Low risk
McAuley, 1985	Low risk	Low risk	High risk	Low risk	High risk	Low risk	Medium risk	Low risk	Low risk	Low risk
McCann et al., 1992	Low risk	Low risk	High risk	Low risk	High risk	Low risk	Medium risk	Low risk	Low risk	Medium risk
Mon-Lopez et al., 2022	Low risk	Low risk	High risk	Low risk	Medium risk	Low risk	Medium risk	Low risk	Medium risk	Medium risk
Papastergiou et al., 2010	Low risk	Low risk	High risk	Low risk	Medium risk	High risk	Medium risk	Low risk	Low risk	Medium risk
Parfitt & Pates, 1999	Low risk	Low risk	High risk	Low risk	Medium risk	Medium risk	High risk	Low risk	Low risk	Low risk
Pinto & Vázquez, 2013	Low risk	Low risk	High risk	Medium risk	Medium risk	Low risk	Medium risk	Low risk	High risk	High risk
Raudsepp & Kais, 2002	Low risk	Low risk	High risk	High risk	Medium risk	Medium risk	Medium risk	Low risk	Low risk	Medium risk
Sanchez et al., 2010	Low risk	Low risk	High risk	Medium risk	Medium risk	Low risk; High risk	Medium risk	Low risk	Medium risk	Medium risk
Silva et al., 2023	Low risk	Low risk	High risk	High risk	Medium risk	High risk	Medium risk	Low risk	Medium risk	Medium risk
Slačanac et al., 2021	Low risk	Low risk	High risk	Low risk	Medium risk	Medium risk	Medium risk	Medium risk	Medium risk	High risk
Swain & Jones, 1996	Low risk	Low risk	High risk	Low risk	Medium risk	Medium risk	Medium risk	Low risk	Low risk	Low risk

Terry, Cox, et al., 1996	Low risk	Low risk	High risk	Medium risk	Medium risk	High risk; High risk	Medium risk	Low risk	Low risk	Medium risk; Medium risk
Terry & Slade, 1995	Low risk	Low risk	High risk	Low risk	Medium risk	High risk	Medium risk	Low risk	Low risk	Medium risk
Tsopani et al., 2011	Low risk	Low risk	High risk	Medium risk	Medium risk	High risk; Medium risk; Low risk	Medium risk	Low risk	High risk	High risk
Van Der Heever et al., 2007	Low risk	Low risk	High risk	Low risk	Medium risk	High risk	Medium risk	Low risk	High risk	High risk
Vlachopoulos et al., 2025	Low risk	Low risk	High risk	Low risk	High risk	High risk; High risk	Medium risk	Low risk	Low risk	High risk
Williams & Krane, 1992	Low risk	Low risk	High risk	Medium risk	High risk	Low risk	Medium risk	High risk	Medium risk	High risk
Zhang et al., 2024	Low risk	Low risk	High risk	Low risk	High risk	Medium risk	Low risk	Low risk	Medium risk	High risk

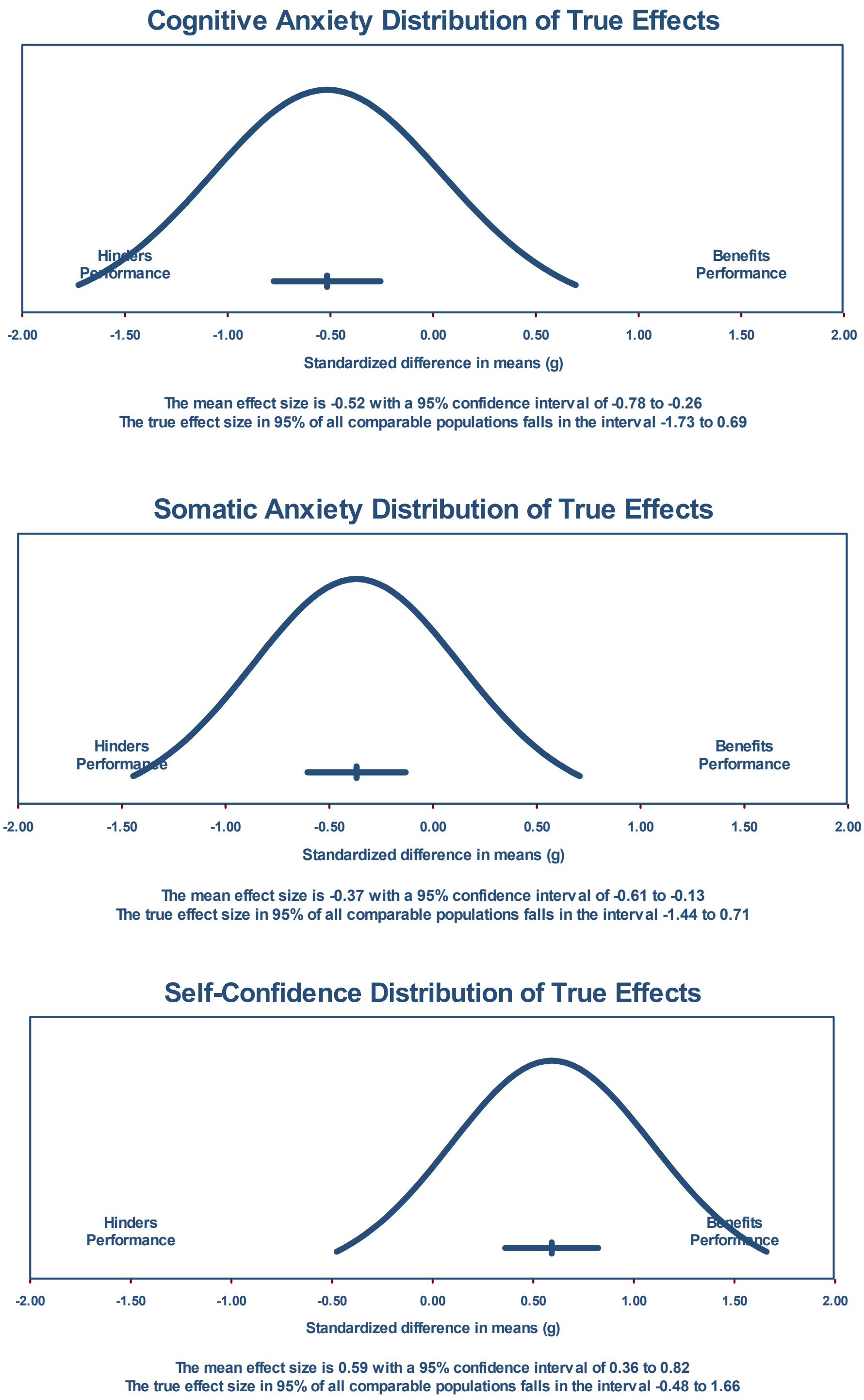

Figure S1. Distribution of true effects for mean difference (*g*) samples. Cognitive anxiety (top), somatic anxiety (middle), and confidence (bottom).

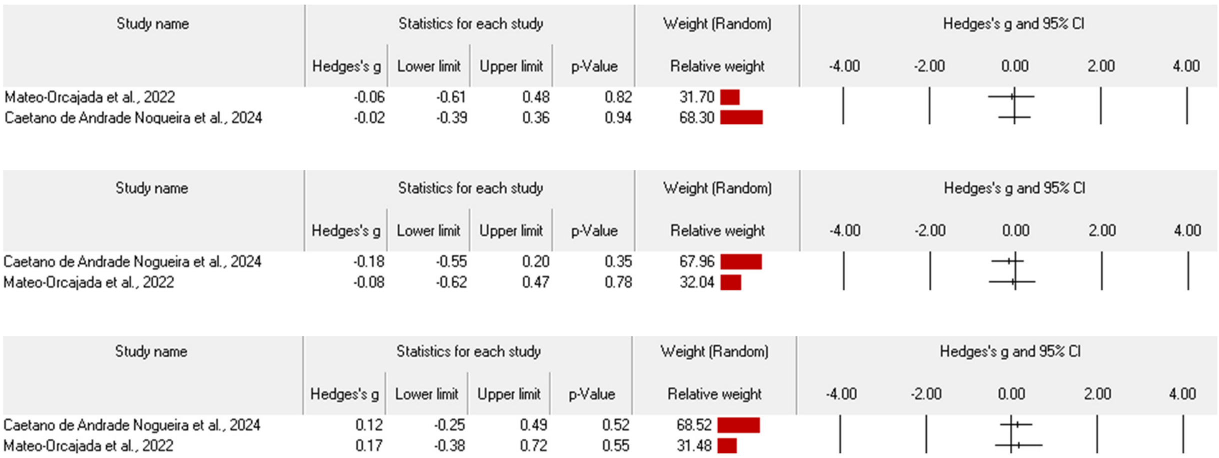

Figure S2. Figures associated with Caetano de Andrade Nogueira et al. [27] and Mateo-Orcajada et al. [45]. Cognitive anxiety (top), somatic anxiety (middle), and confidence (bottom).

Study name	Statistics with study removed			
	Point	Lower limit	Upper limit	p-Value
Aliberti et al., 2024	-0.45	-0.70	-0.20	0.00
Armas Alejo et al., 2020	-0.53	-0.80	-0.26	0.00
Armas Alejo et al., 2025	-0.46	-0.72	-0.20	0.00
Arroyo Del Bosque et al., 2022	-0.53	-0.80	-0.25	0.00
Bois et al., 2009	-0.57	-0.82	-0.31	0.00
Chapman et al., 1997	-0.52	-0.79	-0.24	0.00
Conde-Ripoll et al., 2023	-0.54	-0.81	-0.27	0.00
Conde-Ripoll et al., 2024	-0.54	-0.80	-0.27	0.00
Costa et al., 2019	-0.51	-0.78	-0.25	0.00
Dallas et al., 2019	-0.56	-0.82	-0.29	0.00
Fernandez-Fernandez et al., 2014	-0.47	-0.73	-0.22	0.00
Filaire et al., 2009	-0.50	-0.76	-0.23	0.00
Fuentes-García et al., 2023	-0.54	-0.81	-0.28	0.00
Jones et al., 1993	-0.55	-0.82	-0.29	0.00
Kenshloo et al., 2026	-0.51	-0.77	-0.24	0.00
Krane & Williams, 1994	-0.54	-0.81	-0.27	0.00
Papastergiou et al., 2010	-0.50	-0.77	-0.22	0.00
Sanchez et al., 2010	-0.52	-0.79	-0.25	0.00
Silva et al., 2023	-0.51	-0.78	-0.24	0.00
Terry & Slade, 1995	-0.46	-0.71	-0.22	0.00
Terry et al., 1996	-0.53	-0.80	-0.25	0.00
Tsopani et al., 2011	-0.52	-0.79	-0.26	0.00
Van Der Heever et al., 2007	-0.54	-0.81	-0.27	0.00
Vlachopoulos et al., 2025	-0.50	-0.78	-0.23	0.00

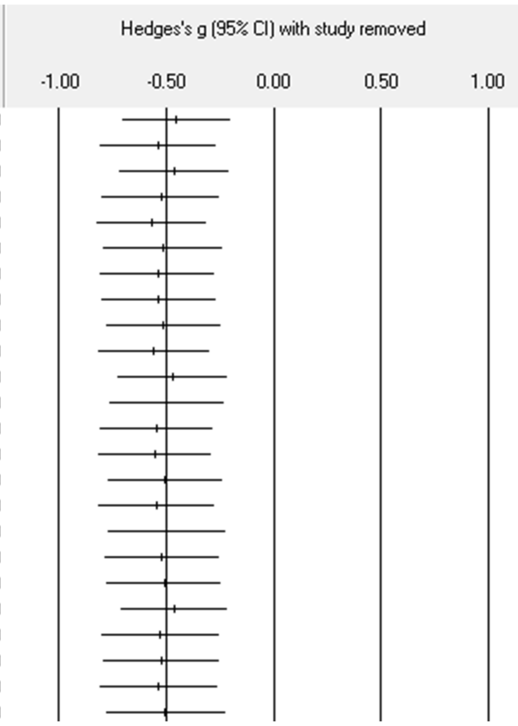

Study name	Statistics with study removed			
	Point	Lower limit	Upper limit	p-Value
Aliberti et al., 2024	-0.35	-0.60	-0.10	0.01
Armas Alejo et al., 2020	-0.38	-0.63	-0.13	0.00
Armas Alejo et al., 2025	-0.31	-0.54	-0.08	0.01
Arroyo Del Bosque et al., 2022	-0.39	-0.64	-0.14	0.00
Bois et al., 2009	-0.42	-0.66	-0.19	0.00
Chapman et al., 1997	-0.37	-0.63	-0.12	0.00
Conde-Ripoll et al., 2023	-0.40	-0.65	-0.16	0.00
Conde-Ripoll et al., 2024	-0.38	-0.63	-0.14	0.00
Costa et al., 2019	-0.38	-0.63	-0.14	0.00
Dallas et al., 2019	-0.38	-0.63	-0.13	0.00
Fernandez-Fernandez et al., 2014	-0.34	-0.58	-0.10	0.01
Filaire et al., 2009	-0.33	-0.57	-0.09	0.01
Fuentes-García et al., 2023	-0.39	-0.64	-0.15	0.00
Jones et al., 1993	-0.40	-0.65	-0.15	0.00
Kenshloo et al., 2026	-0.35	-0.60	-0.11	0.00
Krane & Williams, 1994	-0.39	-0.64	-0.13	0.00
Papastergiou et al., 2010	-0.37	-0.63	-0.11	0.01
Sanchez et al., 2010	-0.41	-0.65	-0.17	0.00
Silva et al., 2023	-0.38	-0.63	-0.14	0.00
Terry & Slade, 1995	-0.30	-0.51	-0.10	0.00
Terry et al., 1996	-0.39	-0.64	-0.13	0.00
Tsopani et al., 2011	-0.39	-0.64	-0.14	0.00
Van Der Heever et al., 2007	-0.40	-0.65	-0.15	0.00
Vlachopoulos et al., 2025	-0.38	-0.64	-0.12	0.00

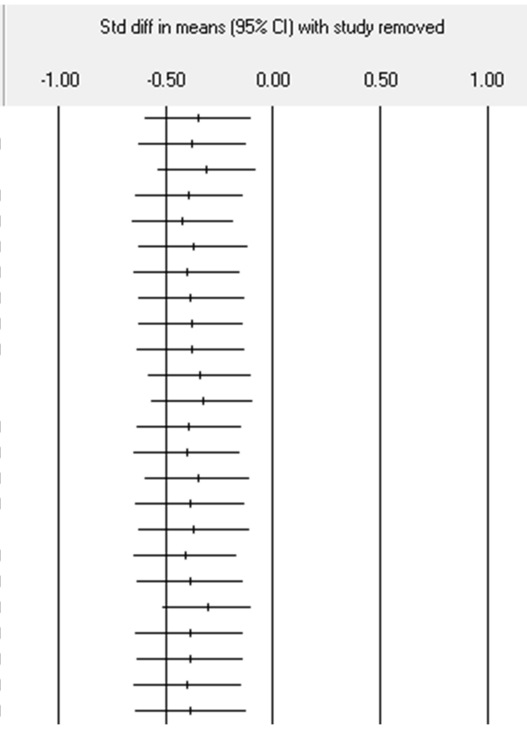

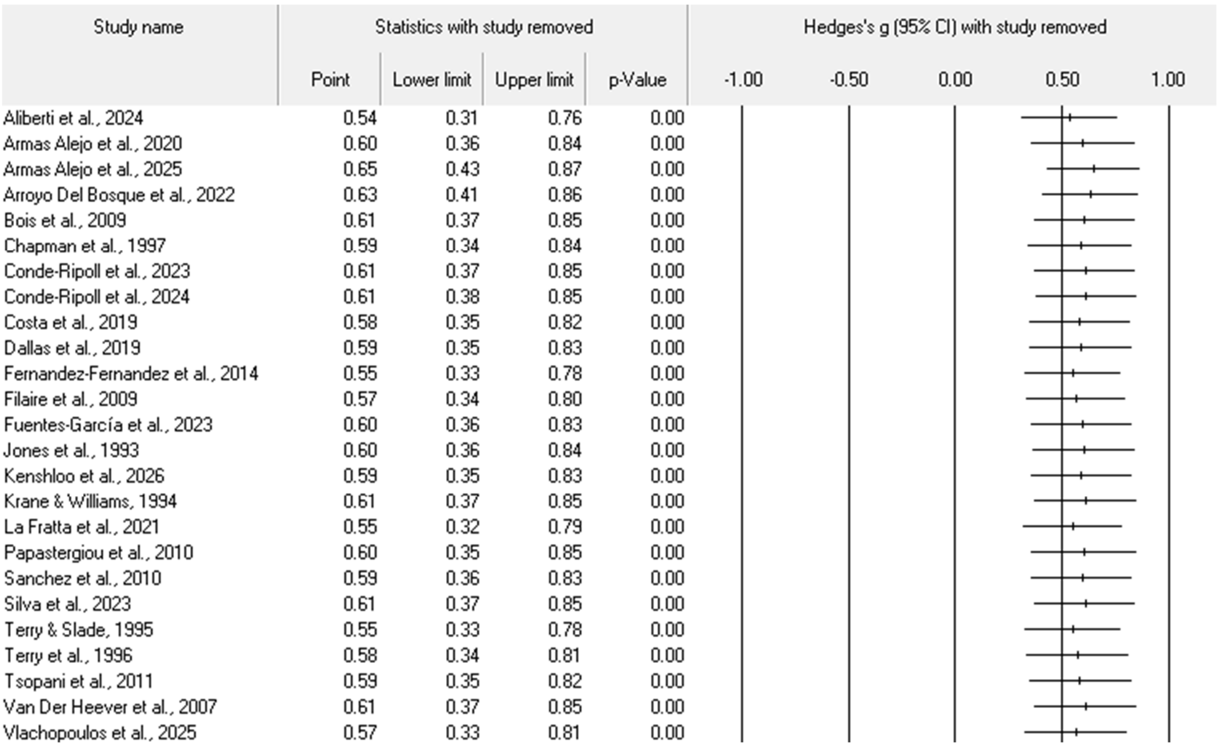

Study name	Statistics with study removed			
	Point	Lower limit	Upper limit	p-Value
Aliberti et al., 2024	0.54	0.31	0.76	0.00
Armas Alejo et al., 2020	0.60	0.36	0.84	0.00
Armas Alejo et al., 2025	0.65	0.43	0.87	0.00
Arroyo Del Bosque et al., 2022	0.63	0.41	0.86	0.00
Bois et al., 2009	0.61	0.37	0.85	0.00
Chapman et al., 1997	0.59	0.34	0.84	0.00
Conde-Ripoll et al., 2023	0.61	0.37	0.85	0.00
Conde-Ripoll et al., 2024	0.61	0.38	0.85	0.00
Costa et al., 2019	0.58	0.35	0.82	0.00
Dallas et al., 2019	0.59	0.35	0.83	0.00
Fernandez-Fernandez et al., 2014	0.55	0.33	0.78	0.00
Filaire et al., 2009	0.57	0.34	0.80	0.00
Fuentes-García et al., 2023	0.60	0.36	0.83	0.00
Jones et al., 1993	0.60	0.36	0.84	0.00
Kenshloo et al., 2026	0.59	0.35	0.83	0.00
Krane & Williams, 1994	0.61	0.37	0.85	0.00
La Fratta et al., 2021	0.55	0.32	0.79	0.00
Papastergiou et al., 2010	0.60	0.35	0.85	0.00
Sanchez et al., 2010	0.59	0.36	0.83	0.00
Silva et al., 2023	0.61	0.37	0.85	0.00
Terry & Slade, 1995	0.55	0.33	0.78	0.00
Terry et al., 1996	0.58	0.34	0.81	0.00
Tsopani et al., 2011	0.59	0.35	0.82	0.00
Van Der Heever et al., 2007	0.61	0.37	0.85	0.00
Vlachopoulos et al., 2025	0.57	0.33	0.81	0.00

Figure S3. One-study removed for mean difference (*g*) samples. Cognitive anxiety studies [18-21,25,27-35,4,39,40,49,53,54,58,59,60,61] (top figure), somatic anxiety studies [18-21,25,27-35,4,39,40,49,53,54,58-61] (middle figure), and self-confidence studies [in order 18-21,25,27-35,4,39-41,49,53,54,58-61] (bottom figure).

Study name	Cumulative statistics			
	Point	Lower limit	Upper limit	p-Value
Jones et al., 1993	0.29	-0.27	0.85	0.31
Krane & Williams, 1994	0.03	-0.26	0.32	0.83
Terry & Slade, 1995	-0.41	-1.47	0.64	0.44
Terry et al., 1996	-0.40	-1.16	0.37	0.31
Chapman et al., 1997	-0.43	-1.01	0.15	0.14
Van Der Heever et al., 2007	-0.38	-0.87	0.10	0.12
Bois et al., 2009	-0.24	-0.72	0.24	0.33
Filaire et al., 2009	-0.31	-0.77	0.15	0.18
Sanchez et al., 2010	-0.32	-0.75	0.11	0.15
Papastergiou et al., 2010	-0.39	-0.78	0.00	0.05
Tsopani et al., 2011	-0.39	-0.75	-0.02	0.04
Fernandez-Fernandez et al., 2014	-0.48	-0.85	-0.11	0.01
Dallas et al., 2019	-0.42	-0.78	-0.05	0.03
Costa et al., 2019	-0.42	-0.77	-0.07	0.02
Armas Alejo et al., 2020	-0.40	-0.73	-0.08	0.02
Arroyo Del Bosque et al., 2022	-0.40	-0.70	-0.10	0.01
Silva et al., 2023	-0.41	-0.71	-0.12	0.01
Conde-Ripoll et al., 2023	-0.39	-0.67	-0.11	0.01
Fuentes-García et al., 2023	-0.36	-0.64	-0.09	0.01
Aliberti et al., 2024	-0.45	-0.73	-0.16	0.00
Conde-Ripoll et al., 2024	-0.43	-0.71	-0.15	0.00
Vlachopoulos et al., 2025	-0.45	-0.71	-0.18	0.00
Armas Alejo et al., 2025	-0.51	-0.77	-0.24	0.00
Kenshloo et al., 2026	-0.52	-0.78	-0.26	0.00

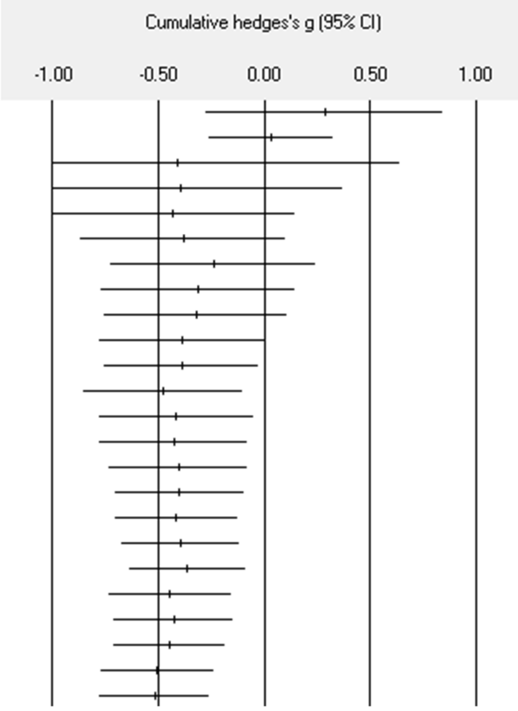

Study name	Cumulative statistics			
	Point	Lower limit	Upper limit	p-Value
Jones et al., 1993	0.17	-0.39	0.72	0.56
Krane & Williams, 1994	-0.09	-0.40	0.22	0.56
Terry & Slade, 1995	-0.50	-1.45	0.46	0.31
Terry et al., 1996	-0.42	-1.14	0.31	0.26
Chapman et al., 1997	-0.43	-0.98	0.11	0.12
Van Der Heever et al., 2007	-0.35	-0.83	0.13	0.16
Bois et al., 2009	-0.20	-0.69	0.28	0.42
Filaire et al., 2009	-0.36	-0.85	0.13	0.15
Papastergiou et al., 2010	-0.37	-0.78	0.04	0.08
Sanchez et al., 2010	-0.28	-0.69	0.13	0.18
Tsopani et al., 2011	-0.26	-0.64	0.12	0.18
Fernandez-Fernandez et al., 2014	-0.34	-0.72	0.04	0.08
Costa et al., 2019	-0.33	-0.69	0.04	0.08
Dallas et al., 2019	-0.32	-0.66	0.02	0.06
Armas Alejo et al., 2020	-0.32	-0.63	-0.01	0.04
Arroyo Del Bosque et al., 2022	-0.30	-0.59	-0.01	0.04
Conde-Ripoll et al., 2023	-0.27	-0.55	0.01	0.06
Fuentes-García et al., 2023	-0.26	-0.53	0.02	0.07
Silva et al., 2023	-0.25	-0.51	0.01	0.06
Aliberti et al., 2024	-0.29	-0.54	-0.03	0.03
Conde-Ripoll et al., 2024	-0.28	-0.53	-0.03	0.03
Armas Alejo et al., 2025	-0.35	-0.61	-0.09	0.01
Vlachopoulos et al., 2025	-0.35	-0.59	-0.11	0.00
Kenshloo et al., 2026	-0.37	-0.61	-0.13	0.00

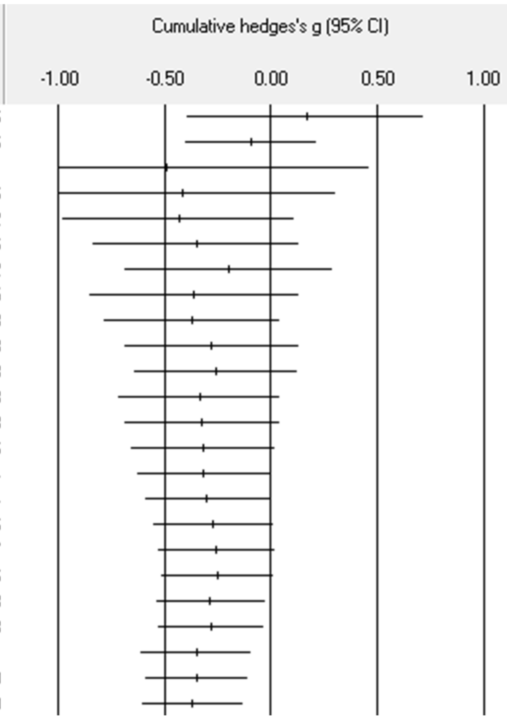

Study name	Cumulative statistics			
	Point	Lower limit	Upper limit	p-Value
Jones et al., 1993	0.38	-0.18	0.94	0.18
Krane & Williams, 1994	0.25	-0.01	0.51	0.06
Terry & Slade, 1995	0.64	-0.13	1.40	0.10
Terry et al., 1996	0.72	0.15	1.29	0.01
Chapman et al., 1997	0.71	0.28	1.14	0.00
Van Der Heever et al., 2007	0.62	0.23	1.01	0.00
Bois et al., 2009	0.57	0.22	0.93	0.00
Filaire et al., 2009	0.63	0.28	0.97	0.00
Sanchez et al., 2010	0.62	0.29	0.95	0.00
Papastergiou et al., 2010	0.59	0.31	0.88	0.00
Tsopani et al., 2011	0.61	0.34	0.87	0.00
Fernandez-Fernandez et al., 2014	0.67	0.39	0.95	0.00
Dallas et al., 2019	0.66	0.40	0.92	0.00
Costa et al., 2019	0.67	0.42	0.92	0.00
Armas Alejo et al., 2020	0.65	0.42	0.88	0.00
La Fratta et al., 2021	0.70	0.47	0.94	0.00
Arroyo Del Bosque et al., 2022	0.64	0.39	0.89	0.00
Silva et al., 2023	0.62	0.37	0.86	0.00
Conde-Ripoll et al., 2023	0.59	0.36	0.82	0.00
Fuentes-García et al., 2023	0.59	0.36	0.81	0.00
Aliberti et al., 2024	0.65	0.41	0.89	0.00
Conde-Ripoll et al., 2024	0.63	0.39	0.86	0.00
Vlachopoulos et al., 2025	0.65	0.42	0.87	0.00
Armas Alejo et al., 2025	0.59	0.35	0.83	0.00
Kenshloo et al., 2026	0.59	0.36	0.82	0.00

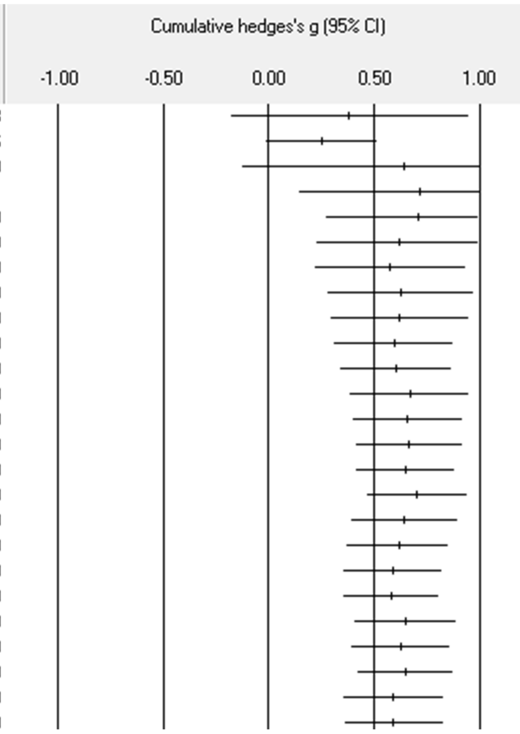

Figure S4. Cumulative analysis by year for mean difference (*g*) samples. Cognitive anxiety studies [18-21,25,27-35,4,39,40,49,53,54,58,59,60,61] (top figure), somatic anxiety studies [18-21,25,27-35,4,39,40,49,53,54,58-61] (middle figure), and self-confidence studies [in order 18-21,25,27-35,4,39-41,49,53,54,58-61] (bottom figure).

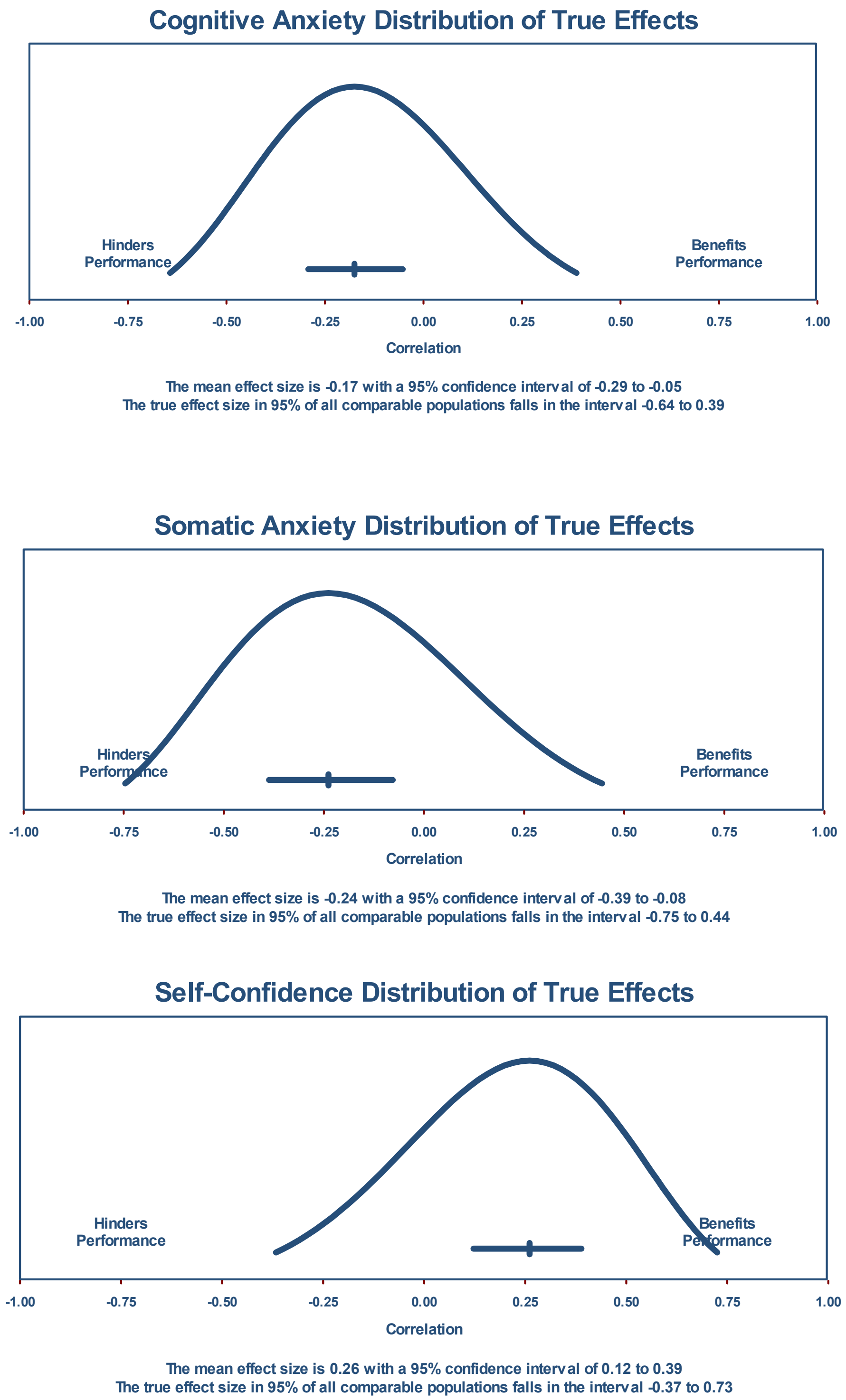

Figure S5. Distribution of true effects for mean difference (*r*) samples. Cognitive anxiety (top), somatic anxiety (middle), and confidence (bottom).

Study name	Statistics with study removed			
	Point	Lower limit	Upper limit	p-Value
Aliberti et al., 2024	-0.13	-0.24	-0.03	0.01
Bois et al., 2009	-0.19	-0.31	-0.07	0.00
Mabweazaa et al., 2016	-0.13	-0.23	-0.02	0.02
Martin & Gill, 1991	-0.19	-0.31	-0.06	0.00
Bejek & Hagtvet, 1996	-0.18	-0.30	-0.06	0.00
Dallas et al., 2019	-0.18	-0.30	-0.05	0.01
Hassmen et al., 2004	-0.17	-0.29	-0.05	0.01
Jones et al., 1993	-0.18	-0.30	-0.06	0.00
Lu et al., 2022	-0.19	-0.31	-0.06	0.00
McAuley, 1985	-0.18	-0.29	-0.05	0.01
Mon-Lopez et al., 2022	-0.18	-0.30	-0.05	0.01
Pinto & Vázquez, 2013	-0.18	-0.30	-0.05	0.01
Sanchez et al., 2010	-0.19	-0.30	-0.06	0.00
Tsopani et al., 2011	-0.19	-0.31	-0.06	0.00
Williams & Krane, 1992	-0.17	-0.30	-0.04	0.01
Costa et al., 2019	-0.17	-0.29	-0.05	0.01
Kais & Raudsepp, 2004	-0.18	-0.30	-0.05	0.01
León-Prados et al., 2014 Study 1	-0.17	-0.29	-0.05	0.01
León-Prados et al., 2014 Study 2	-0.17	-0.29	-0.05	0.01
Parfitt & Pates, 1999	-0.17	-0.29	-0.05	0.01
Raudsepp & Kais, 2002	-0.19	-0.31	-0.06	0.00
Swain & Jones, 1996	-0.18	-0.29	-0.05	0.01
Zhang et al., 2024	-0.17	-0.30	-0.04	0.01
Beattie et al., 2004	-0.19	-0.31	-0.07	0.00
Heazlewood & Burke, 2011	-0.18	-0.31	-0.04	0.01
McCann et al., 1992	-0.17	-0.29	-0.04	0.01
Barnes et al., 1986	-0.17	-0.29	-0.04	0.01
Burton, 1988 Study 1	-0.16	-0.28	-0.04	0.01
Burton, 1988 Study 2	-0.18	-0.30	-0.05	0.01

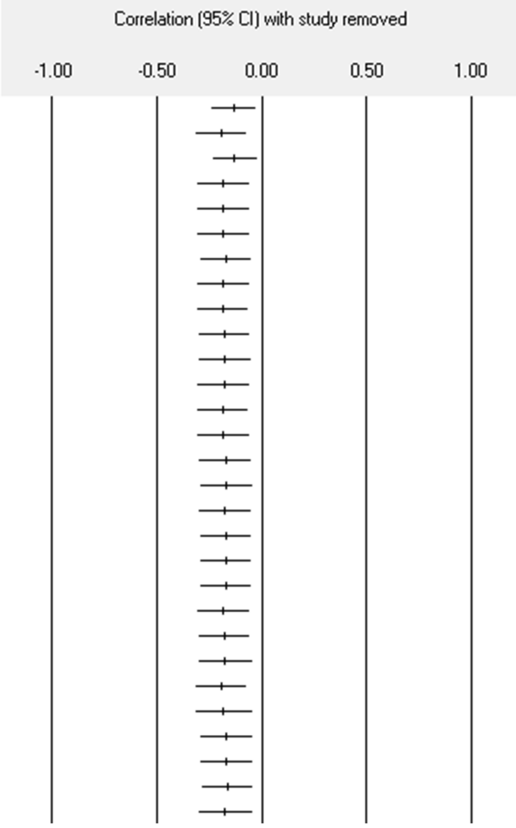

Study name	Statistics with study removed			
	Point	Lower limit	Upper limit	p-Value
Aliberti et al., 2024	-0.22	-0.37	-0.05	0.01
Bois et al., 2009	-0.26	-0.41	-0.10	0.00
Mabweazaa et al., 2016	-0.17	-0.29	-0.05	0.01
Dallas et al., 2019	-0.24	-0.40	-0.07	0.01
Hassmen et al., 2004	-0.23	-0.38	-0.07	0.01
Jones et al., 1993	-0.26	-0.41	-0.09	0.00
Lu et al., 2022	-0.21	-0.37	-0.05	0.01
McAuley, 1985	-0.24	-0.39	-0.08	0.00
Mon-Lopez et al., 2022	-0.24	-0.39	-0.07	0.01
Pinto & Vázquez, 2013	-0.25	-0.40	-0.08	0.00
Sanchez et al., 2010	-0.26	-0.41	-0.10	0.00
Tsopani et al., 2011	-0.26	-0.41	-0.09	0.00
Costa et al., 2019	-0.24	-0.40	-0.08	0.00
Kais & Raudsepp, 2004	-0.24	-0.40	-0.07	0.01
Parfitt & Pates, 1999	-0.23	-0.39	-0.07	0.01
Raudsepp & Kais, 2002	-0.25	-0.40	-0.08	0.00
Swain & Jones, 1996	-0.25	-0.40	-0.08	0.00
Heazlewood & Burke, 2011	-0.23	-0.40	-0.04	0.02
McCann et al., 1992	-0.22	-0.38	-0.06	0.01
Barnes et al., 1986	-0.24	-0.39	-0.08	0.00
Burton, 1988 Study 1	-0.23	-0.39	-0.07	0.01
Burton, 1988 Study 2	-0.25	-0.41	-0.08	0.00

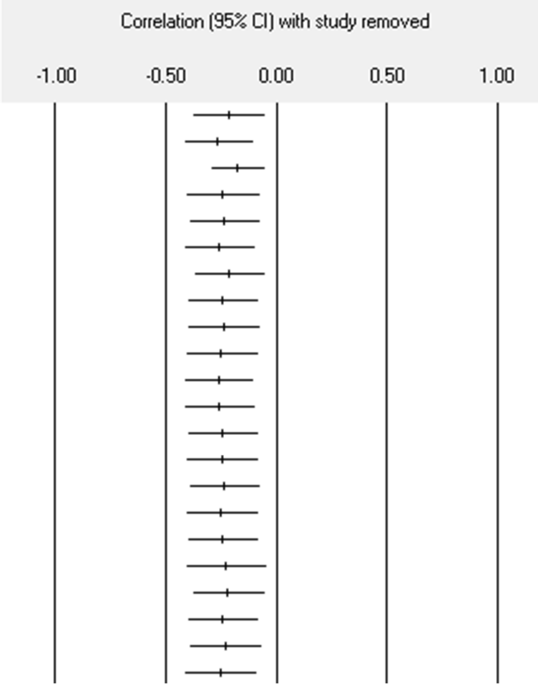

Study name	Statistics with study removed			
	Point	Lower limit	Upper limit	p-Value
Aliberti et al., 2024	0.22	0.10	0.33	0.00
Bois et al., 2009	0.26	0.12	0.39	0.00
Slacanac et al., 2021	0.27	0.12	0.41	0.00
Bejek & Hagtvet, 1996	0.27	0.13	0.41	0.00
Dallas et al., 2019	0.26	0.12	0.40	0.00
Hassmen et al., 2004	0.25	0.11	0.38	0.00
Jones et al., 1993	0.26	0.12	0.39	0.00
Lu et al., 2022	0.26	0.12	0.40	0.00
McAuley, 1985	0.26	0.12	0.39	0.00
Mon-Lopez et al. 2022	0.27	0.13	0.40	0.00
Pinto & Vázquez, 2013	0.26	0.11	0.39	0.00
Sanchez et al., 2010	0.28	0.14	0.41	0.00
Tsopani et al., 2011	0.26	0.11	0.39	0.00
Costa et al., 2019	0.27	0.13	0.40	0.00
Kais & Raudsepp, 2004	0.25	0.11	0.38	0.00
León-Prados et al., 2014	0.26	0.12	0.39	0.00
Parfitt & Pates, 1999	0.24	0.10	0.37	0.00
Raudsepp & Kais, 2002	0.25	0.11	0.38	0.00
Swain & Jones, 1996	0.26	0.12	0.39	0.00
Beattie et al., 2004	0.28	0.13	0.41	0.00
Heazlewood & Burke, 2011	0.28	0.17	0.39	0.00
McCann et al., 1992	0.26	0.11	0.39	0.00
Barnes et al., 1986	0.26	0.12	0.39	0.00
Burton, 1988 Study 1	0.25	0.11	0.39	0.00
Burton, 1988 Study 2	0.27	0.12	0.41	0.00

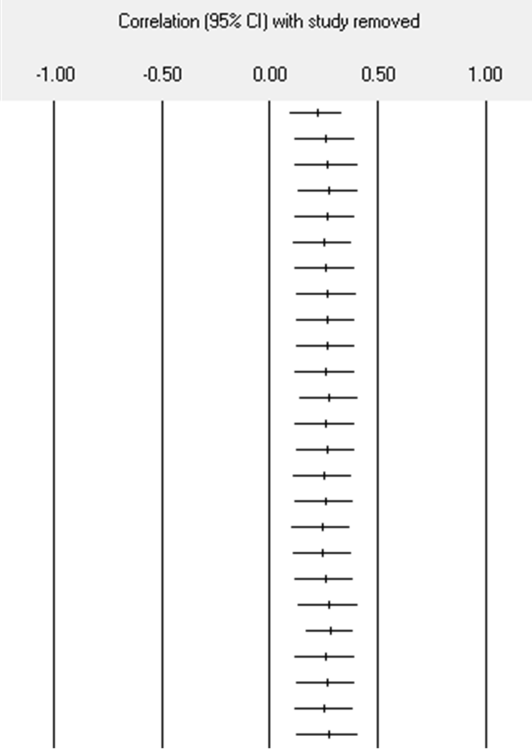

Figure S6. One-study removed for mean difference (*r*) samples. Cognitive anxiety studies [in order 20,22-26,31,32,36-38,12,42-44,46-48,50-53,56,59,62,63] (top figure), somatic anxiety studies [in order 20,22,25,26,31,32,36-38,42,43,46-48,50-53,56,59] (middle figure), and self-confidence studies [20,22-26,31,32,4,38,12,42,46-48,50-53,55,56,59].

Study name	Cumulative statistics			
	Point	Lower limit	Upper limit	p-Value
McAuley, 1985	-0.11	-0.80	0.70	0.83
Barnes et al., 1986	-0.32	-0.68	0.17	0.20
Burton, 1988 Study 1	-0.42	-0.66	-0.11	0.01
Burton, 1988 Study 2	-0.23	-0.38	-0.07	0.01
Martin & Gill, 1991	-0.17	-0.38	0.06	0.15
Williams & Krane, 1992	-0.18	-0.34	-0.01	0.04
McCann et al., 1992	-0.21	-0.36	-0.04	0.01
Jones et al., 1993	-0.17	-0.31	-0.03	0.02
Bejek & Hagtvet, 1996	-0.15	-0.27	-0.01	0.03
Swain & Jones, 1996	-0.14	-0.26	-0.02	0.02
Parfitt & Pates, 1999	-0.15	-0.27	-0.03	0.01
Raudsepp & Kais, 2002	-0.13	-0.24	-0.01	0.03
Hassmen et al., 2004	-0.13	-0.24	-0.02	0.02
Kais & Raudsepp, 2004	-0.13	-0.23	-0.03	0.01
Beattie et al., 2004	-0.11	-0.21	0.01	0.07
Bois et al., 2009	-0.08	-0.20	0.04	0.18
Sanchez et al., 2010	-0.07	-0.18	0.05	0.24
Tsopani et al., 2011	-0.05	-0.16	0.05	0.31
Heazlewood & Burke, 2011	-0.06	-0.15	0.04	0.24
Pinto & Vázquez, 2013	-0.06	-0.14	0.03	0.18
León-Prados et al., 2014	-0.06	-0.15	0.02	0.14
León-Prados et al., 2014	-0.07	-0.15	0.02	0.12
Mabweazaa et al., 2016	-0.15	-0.27	-0.02	0.02
Dallas et al., 2019	-0.14	-0.26	-0.02	0.02
Costa et al., 2019	-0.14	-0.26	-0.03	0.02
Lu et al., 2022	-0.13	-0.25	-0.02	0.02
Mon-Lopez et al., 2022	-0.13	-0.24	-0.02	0.02
Aliberti et al., 2024	-0.17	-0.30	-0.04	0.01
Zhang et al., 2024	-0.17	-0.29	-0.05	0.01

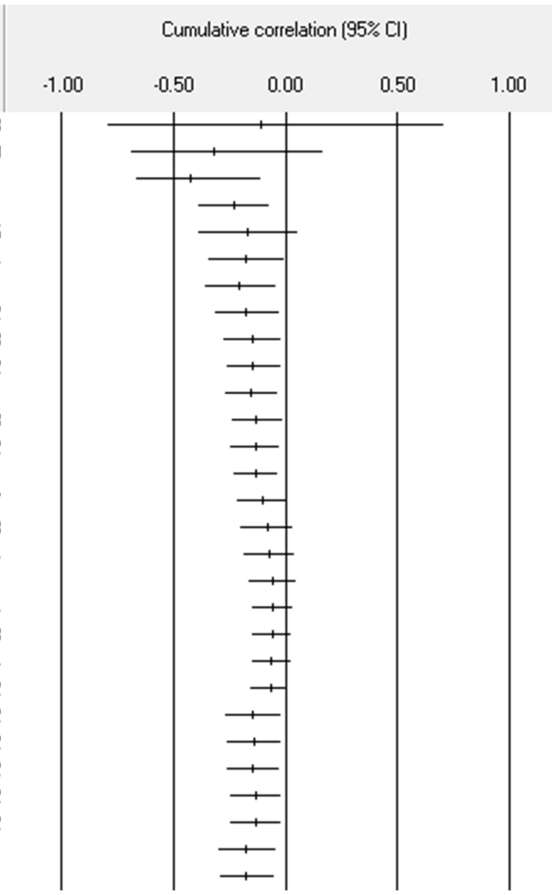

Study name	Cumulative statistics			
	Point	Lower limit	Upper limit	p-Value
McAuley, 1985	0.01	-0.75	0.76	0.99
Barnes et al., 1986	-0.12	-0.56	0.37	0.64
Burton, 1988 Study 1	-0.27	-0.55	0.07	0.12
Burton, 1988 Study 2	-0.09	-0.25	0.07	0.28
McCann et al., 1992	-0.24	-0.48	0.02	0.07
Jones et al., 1993	-0.16	-0.37	0.07	0.17
Swain & Jones, 1996	-0.14	-0.34	0.07	0.19
Parfitt & Pates, 1999	-0.16	-0.34	0.04	0.12
Raudsepp & Kais, 2002	-0.13	-0.28	0.03	0.12
Hassmen et al., 2004	-0.13	-0.28	0.02	0.09
Kais & Raudsepp, 2004	-0.12	-0.25	0.00	0.05
Bois et al., 2009	-0.09	-0.23	0.05	0.19
Sanchez et al., 2010	-0.07	-0.21	0.07	0.31
Tsopani et al., 2011	-0.05	-0.17	0.07	0.43
Heazlewood & Burke,	-0.10	-0.24	0.05	0.18
Pinto & Vázquez, 2013	-0.09	-0.22	0.04	0.16
Mabweazaa et al., 2016	-0.19	-0.37	0.01	0.06
Dallas et al., 2019	-0.19	-0.36	-0.01	0.04
Costa et al., 2019	-0.18	-0.35	-0.01	0.04
Lu et al., 2022	-0.21	-0.37	-0.04	0.02
Mon-Lopez et al., 2022	-0.22	-0.37	-0.05	0.01
Aliberti et al., 2024	-0.24	-0.39	-0.08	0.00

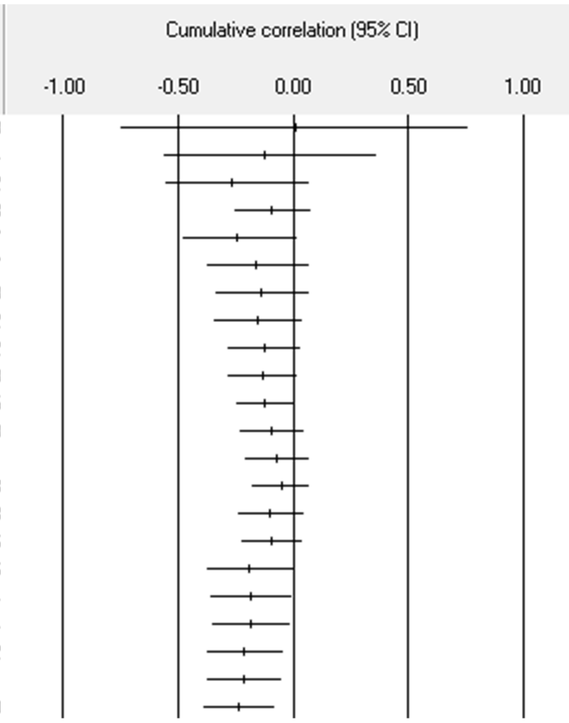

Study name	Cumulative statistics			
	Point	Lower limit	Upper limit	p-Value
McAuley, 1985	0.01	-0.75	0.76	0.99
Barnes et al., 1986	0.14	-0.35	0.57	0.58
Burton, 1988 Study 1	0.30	-0.04	0.57	0.08
Burton, 1988 Study 2	0.13	-0.04	0.29	0.12
McCann et al., 1992	0.16	0.01	0.31	0.04
Jones et al., 1993	0.19	0.06	0.32	0.01
Bejek & Hagtvet, 1996	0.14	0.03	0.26	0.02
Swain & Jones, 1996	0.15	0.03	0.26	0.01
Parfitt & Pates, 1999	0.23	0.07	0.38	0.01
Raudsepp & Kais, 2002	0.29	0.12	0.44	0.00
Hassmen et al., 2004	0.31	0.14	0.46	0.00
Kais & Raudsepp, 2004	0.33	0.18	0.47	0.00
Beattie et al., 2004	0.30	0.14	0.44	0.00
Bois et al., 2009	0.29	0.14	0.42	0.00
Sanchez et al., 2010	0.26	0.12	0.40	0.00
Tsopani et al., 2011	0.26	0.13	0.39	0.00
Heazlewood & Burke, 2011	0.24	0.07	0.39	0.01
Pinto & Vázquez, 2013	0.24	0.08	0.39	0.00
León-Prados et al., 2014	0.24	0.09	0.39	0.00
Dallas et al., 2019	0.24	0.10	0.38	0.00
Costa et al., 2019	0.23	0.09	0.37	0.00
Slacanac et al., 2021	0.23	0.10	0.35	0.00
Lu et al., 2022	0.22	0.10	0.34	0.00
Mon-Lopez et al. 2022	0.22	0.10	0.33	0.00
Aliberti et al., 2024	0.26	0.12	0.39	0.00

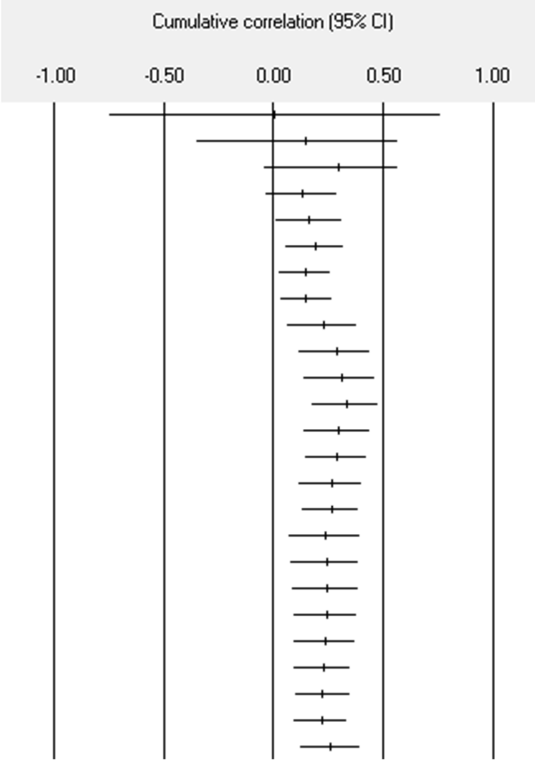

Figure S7. Cumulative analysis by year for mean difference (*r*) samples. Cognitive anxiety studies [in order 20,22-26,31,32,36-38,12,42-44,46-48,50-53,56,59,62,63] (top figure), somatic anxiety studies [in order 20,22,25,26,31,32,36-38,42,43,46-48,50-53,56,59] (middle figure), and self-confidence studies [20,22-26,31,32,4,38,12,42,46-48,50-53,55,56,59].

Report S1. Effect size, odds ratio conversion, and interpretation: Self-confidence winners vs. losers

Effect Size, Odds Ratio Conversion, and Interpretation

Self-confidence: winners vs. losers

The data

Two groups, *winners* and *losers*, with mean self-confidence scores:

Group	Mean	SD
Winners	23.96	5.79
Losers	20.51	4.61

Group sizes were not specified, so the calculations below assume **equal n** — the only case in which the pooled SD reduces to a simple average of the two variances. With unequal groups the pooled SD must be weighted by the degrees of freedom (see note at the end).

Step 1 — Cohen's d

The standardized mean difference is the raw difference divided by the pooled standard deviation:

$$d = (M_1 - M_2) / SD_{\text{pooleə}}$$

With equal n, the pooled SD is:

$$SD_{\text{pooleə}} = \sqrt{[(SD_1^2 + SD_2^2) / 2]}$$

Substituting:

$$SD_{\text{pooleə}} = \sqrt{[(5.79^2 + 4.61^2) / 2]} = \sqrt{[(33.524 + 21.252) / 2]} = \sqrt{27.388} = 5.233$$

$$d = (23.96 - 20.51) / 5.233 = 3.45 / 5.233$$

$$\mathbf{d \approx 0.66}$$

Step 2 — Converting d to an odds ratio

The standard conversion (Hasselblad & Hedges) assumes the underlying continuous outcome follows a *logistic* distribution, and maps *d* to the log odds ratio:

$$\ln(OR) = d \times \pi/\sqrt{3} = d \times 1.8138$$

Substituting:

$$\ln(OR) = 0.659 \times 1.8138 = 1.195$$

$$OR = e^{1.195}$$

OR ≈ 3.30

Step 3 — Interpreting the odds ratio

This requires dichotomizing the continuous self-confidence score into "high" vs. "low" (above/below some threshold). The odds ratio then says:

The odds of being in the high-confidence category are about 3.3 times greater for winners than for losers.

Equivalently, with a cutpoint at the losers' median you would expect roughly 50% of losers above it versus about 77% of winners — odds of 0.77/0.23 ≈ 3.3 versus 0.50/0.50 = 1.0, giving the ratio of 3.3.

Two cautions

Model-dependent. The conversion presumes logistic latent distributions, and the resulting OR is an *approximation* to what an actual dichotomization would give, not an exact equivalent.

Cutpoint sensitivity. The OR is invariant to where you cut *only* under the logistic assumption. In real data the observed OR shifts with the cutpoint, so the single value above is best read as a standardized summary rather than a prediction for any particular threshold.

Step 4 — Reversing the direction: "likelihood of winning"

A natural follow-up is to flip the statement around: "How much more likely are above-median people to win than below-median people?" The obstacle here is not algebraic — it is *directional*.

The OR is symmetric as a *measure of association*, but not as a *predictive statement* about a fixed group. Going from "confidence given outcome" to "winning given confidence" requires the overall base rate of winning, which never entered the *d* calculation. By Bayes, P(win | high) depends on the marginal probability of winning, so two datasets with identical *d* can yield very different "odds of winning given high confidence" depending on whether winning is common or rare.

What transfers cleanly

The odds ratio itself **is** invariant to the direction of conditioning (unlike a risk ratio). So 3.30 is legitimately interpretable as:

The odds of winning are about 3.3 times higher for someone above the confidence median than for someone below it — provided "above/below median" partitions the combined sample 50/50, which is what "median" implies.

What does not transfer without the base rate

You cannot recover the *probability* (as opposed to the odds) of winning in each group without knowing the overall probability of winning, *p*. Only with *p* pinned down do the two group probabilities become

identified. With $p = 0.5$ (equal winners and losers, which this setup implies), the median split gives odds of about 1.82 vs. 0.55, i.e.:

about 65% of above-median people are winners vs. about 35% of below-median people — mirroring the 77/50 figures above, now read in the predictive direction.

This probability statement (65% vs. 35%) is valid only under the equal-group, logistic-latent assumptions used throughout, and shifts as soon as the real base rate of winning departs from 0.5. The odds-ratio statement, by contrast, needs nothing extra.

Notes

Unequal group sizes. If the groups are not equal, replace the pooled SD with the df-weighted version:

$$SD_{\text{pooled}} = \sqrt{[((n_1-1)\cdot SD_1^2 + (n_2-1)\cdot SD_2^2) / (n_1 + n_2 - 2)]}$$

Carrying the variance through. If this is feeding a meta-analysis that pools d-based and OR-based studies on a common scale, the $d \rightarrow \ln(OR)$ direction is the usual move, and the variance transforms as:

$$\text{Var}(\ln OR) = \text{Var}(d) \times (\pi^2/3) = \text{Var}(d) \times 3.2899$$

Table S5. L. James and B. Steward data sets.

	d value			
	0.2	0.5	0.8	1.0
L. James Points/40 min across 23 yr career	+0.48	+1.20	+1.93	2.41
L. James Rebounds/40 min across 23 yr career	+0.22	+0.55	+0.88	1.10
L. James Assists/40 min across 23 yr career	+0.29	+0.74	+1.18	1.48
B. Stewart FG% across 10 yr career	+0.50%	+1.25%	+2.0%	2.5%
B. Stewart FT% across 10 yr career	+0.62%	+1.55%	+2.48%	3.1%

L. James Career Statistics

Descriptive Statistics

	N	Minimum	Maximum	Mean	Std. Deviation
PTS.per.40min	23	21	33	28.65	2.419
RBS.per.40min	23	5.50	9.60	8.0435	1.10488
ASTS.per.40min	23	5.90	11.80	8.0043	1.48124
Valid N (listwise)	23				

B. Stewart WNBA Career

Descriptive Statistics

	N	Minimum	Maximum	Mean	Std. Deviation
BS.FG.Percentage	10	43.90	52.90	46.4900	2.53309
BS.FT.Percentage	10	78.70	89.40	84.4100	3.11250
Valid N (listwise)	10				
